# Stress responses to repeated captures in a wild ungulate

**DOI:** 10.1038/s41598-022-20270-z

**Published:** 2022-09-29

**Authors:** L. Monica Trondrud, Cassandra Ugland, Erik Ropstad, Leif Egil Loe, Steve Albon, Audun Stien, Alina L. Evans, Per Medbøe Thorsby, Vebjørn Veiberg, R. Justin Irvine, Gabriel Pigeon

**Affiliations:** 1 Faculty of Environmental Sciences and Natural Resource Management, Norwegian University of Life Sciences, NO-1432 Ås, Norway; 2 Centre for Biodiversity Dynamics, Institute of Biology, NTNU, NO-7491 Trondheim, Norway; 3 Faculty of Veterinary Science, University of Life Sciences, NO-1432 Ås, Norway; 4 The James Hutton Institute, Craigiebuckler, Aberdeen, AB15 8QH UK; 5 Department of Arctic and Marine Biology, The Arctic University of Norway, NO-9037 Tromsø, Norway; 6 Department of Forestry and Wildlife Management, Inland Norway University of Applied Sciences, Campus Evenstad, NO-2406 Elverum, Norway; 7 Hormone Laboratory, Department of Medical Biochemistry and Biochemical Endocrinology and Metabolism Research Group, Oslo University Hospital, Aker, Oslo, Norway; 8 Department of Terrestrial Ecology, Norwegian Institute for Nature Research, Torgarden, P.O. Box 5685, NO-7485 Trondheim, Norway; 9 Frankfurt Zoological Society, South Africa Street, P.O Box 100003, Addis Ababa, Ethiopia; 10 Institut de Recherche Sur Les Forêts, Université du Québec en Abitibi-Témiscamingue, Rouyn-Noranda, QC J9X 5E4 Canada

**Keywords:** Behavioural ecology, Ecophysiology, Population dynamics

## Abstract

While capture-mark-recapture studies provide essential individual-level data in ecology, repeated captures and handling may impact animal welfare and cause scientific bias. Evaluating the consequences of invasive methodologies should be an integral part of any study involving capture of live animals. We investigated short- and long-term stress responses to repeated captures within a winter on the physiology, behaviour, and reproductive success of female Svalbard reindeer (*Rangifer tarandus platyrhynchus*). Short-term responses were evaluated using serum concentrations of glucocorticoids and catecholamines during handling, and post-release recovery times in heart rate and activity levels. Repeated captures were associated with an increase in measured catecholamines and glucocorticoids, except cortisone, and delayed recovery in heart rate but not activity. Four months later, in summer, individuals captured repeatedly in winter exhibited a small increase in behavioural response to human disturbance and had a lower probability of being observed with a calf, compared to animals not captured, or captured only once. Our findings imply that single annual capture events have no significant negative consequences for Svalbard reindeer, but repeated captures within a season may impact offspring survival in the same year. Such unanticipated side effects highlight the importance of addressing multiple indicators of animal responses to repeated captures.

## Introduction

Ecological and physiological studies of wild animals often depend on capturing and marking individuals^1^. Different scientific questions demand different capture frequencies, from once per lifetime for studies of dispersal^2^, to repeated captures over days or weeks to measure energy expenditure^3^, which may involve the deployment, and later retrieval, of biologging devices^4^. However, handling wild animals and undertaking invasive procedures may induce stress, which can negatively impact the animals’ physiology, behaviour and movement patterns, and potentially influence their reproductive success and survival^5^.

Stress responses to capture can include immediate and/or short-term consequences, like physical injury^6^ or neuroendocrine responses^6,7^, while potential consequences for reproduction and survival may only be apparent over longer, annual, time scales^8,9^. Evaluating only one time scale may not provide a full picture, yet studies combining the short-term (days to weeks) effects of capture on physiological and behavioural measures, and their long-term (months to years) individual and demographic consequences, are uncommon (but see^8^). Such information is key to understanding which biological processes can be investigated in a study population without compromising animal welfare, or potentially introducing scientific bias^10^.

Short-term physiological responses to capture and handling can provide insight into the level of stress that individuals experience during such events. When threatened, animals may enter a state of hyperarousal (“acute stress response”)^11^. This response is driven by the rapid release of catecholamines (adrenaline and noradrenaline) into the bloodstream, followed by the synthesis and release of glucocorticoid hormones, cortisol and related metabolites, by the hypothalamic–pituitary–adrenal (HPA) axis^12^. In turn, this leads to a multitude of physiological responses, including increased heart rate (HR), blood pressure, and body temperature^13^. These responses have been recorded in conjunction with human disturbance in a variety of wild animals^8,9,14–17^.

The use of sedatives for chemical immobilization during capture and handling are often necessary for the safety of both animal and researcher, and sedation can reduce the animal’s perception of stressful or painful stimuli^18^. Some sedative drugs, such as opioids and alpha-adrenoceptor (α2) agonists can inhibit, slow, or even exacerbate the release of glucocorticoids, and their effects vary greatly among different species^18–21^. Delayed effects of sedation, such as slowed movement or altered behaviour could slow post-capture recovery rates^22^. Further, some α2 agonists have been reported to negatively affect the foetal development ^23,24^, and reproductive success in some ungulates^25,26^, but not others^27,28^, indicating that implications of using sedatives during handling are complex, and should be evaluated at the species level^18^.

The acute stress response is an essential mechanism to survive a stressor^12^, and is considered an adaptive function in wild animals^29^. Fluctuations in glucocorticoid levels occur naturally, on both a daily and seasonal scale^30^, which enable animals to perform strenuous activities such as mating, resource competition and predator avoidance^29^. However, repeated negative stimuli, such as capture events, could cause “maladaptive” chronic stress, potentially resulting in altered behaviour^31^, reductions in body condition^8^ and immune system function^32^, with negative implications for both reproduction and survival^33^. For instance, capture events have been associated with short term changes in daily rhythms in roe deer (*Capreolus capreolus*)^34^ and movement patterns in white-tailed deer (*Odocoileus virginianus*)^35^. Further, repeated capture events have been associated with long-term reductions in body condition in both grizzly (*Ursus arctos*) and American black bears (*U. americanus*)^8^, although not in polar bears (*U. maritimus*)^22^. Also, repeated captures over the lifetime have been found to reduce reproductive output in Eurasian beavers (*Castor fiber*)^9^, but not in Alpine ibex (*Capra ibex*)^28^. If a high proportion of individuals exhibit reduced fecundity and/or survival due to capture and handling events, individual-level effects may influence population-level processes^36^.

**Figure 1:**
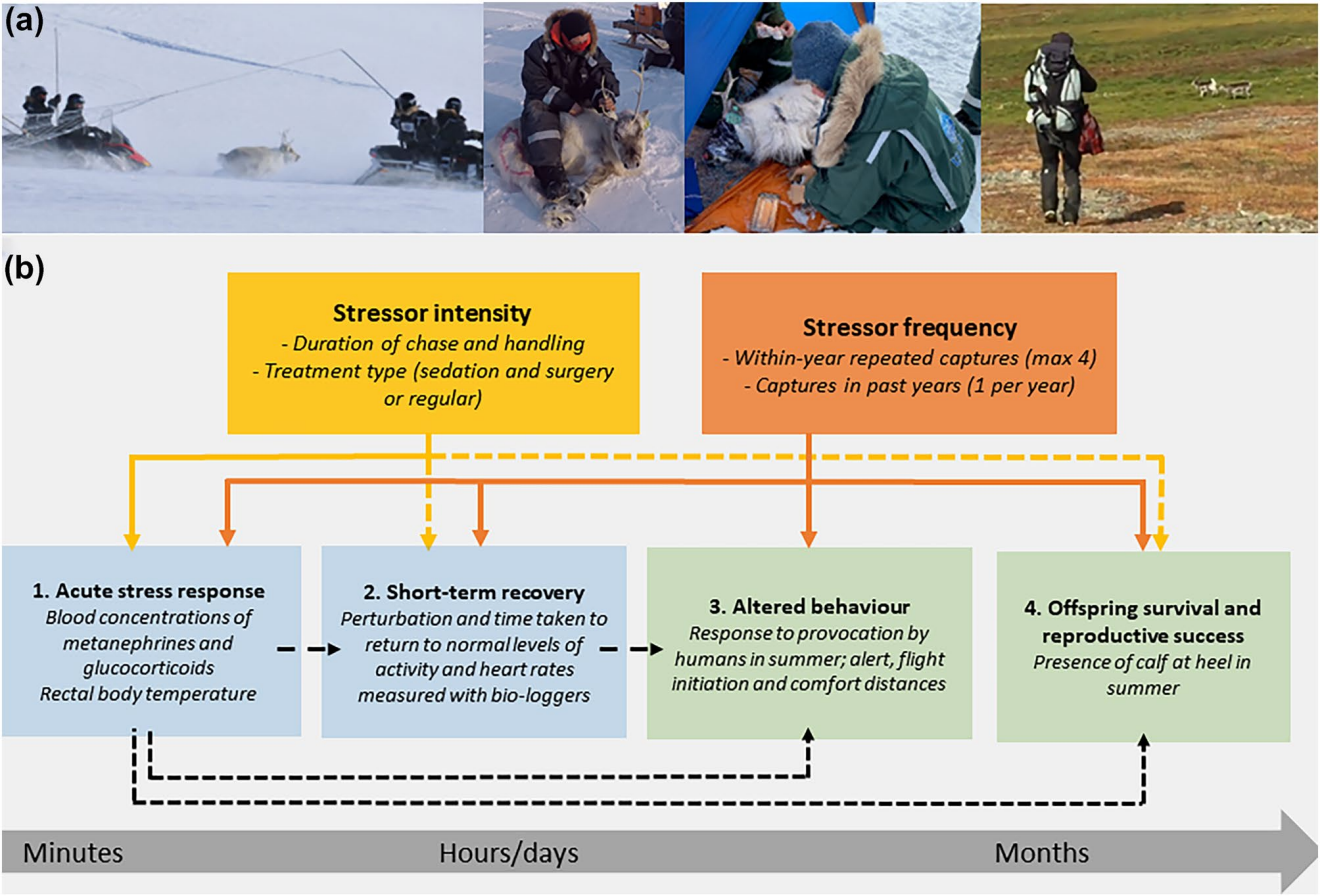
Conceptual figure of the study. Panel (**a**) shows photos from field work, all taken by Erik Ropstad. From left to right: capture of reindeer using a net held between two snowmobiles; manual restraint of reindeer; surgery performed on sedated reindeer; provocation of reindeer by approaching observer. Panel (**b**) provides an overview of the study and data collected. The first row of boxes indicate which type of potential stressors were evaluated, while the second row summarises the responses measured in winter (blue) and in summer (green). Arrows indicate which relationships were tested, with solid and dashed lines indicating significant and non-significant results, respectively.

Previously, we have used our long-term capture-mark-recapture study of Svalbard reindeer (*Rangifer tarandus platyrhynchus*) on Nordenskiöld Land, Spitsbergen^37^ to evaluate the effect of a single annual capture event. We found that one capture per winter season elicited an acute stress response, but no longer-term consequences on reproduction and survival^14^. In 2018, some females were captured up to four times within a six-week period, including a surgical procedure involving sedation^3^. Monitoring many more parameters than in our first study, we use this opportunity to evaluate the consequences of an intensively repeated capture regime. Our approach quantified the effects of stressor intensity (duration of chase, handling time, and surgical procedure involving sedation), as well as stressor frequency (repeated captures), on the acute stress response, the short-term recovery in heart rate (HR) and activity levels, behavioural responses, and reproductive success later in the year (see Fig. 1 for a conceptualization of the study). We predicted that capture events would elicit an acute stress response in the reindeer, involving increases in serum catecholamines, glucocorticoids and body temperature in direct response to stressor intensity^14^ (Prediction, P1.1), but that these responses would be reduced in sedated animals (P1.2). We also predicted that the acute stress response would be stronger with increasing stressor frequency^11^ (P1.3), and animals would exhibit longer post-capture recovery time in both activity levels (P2.1) and HR (P2.2) with each repeated capture. Furthermore, we predicted that repeatedly captured individuals would lose mass at a higher rate than the expected seasonal loss in individuals captured once (P3.1). In turn, this higher mass loss would be expected to reduce reproductive success (P3.2). Lastly, we predicted that repeatedly captured individuals would display greater alertness- and flight behaviour, four months later, in summer (P4). By using different physiological, behavioural, and reproductive measures we provide a rare, integrative assessment of the effects of capture intensity and frequency in a wild animal. Understanding how animals respond to methods and frequencies of invasive data collection can inform which biological processes can, and cannot, be studied without introducing scientific bias and/or compromising individual animal welfare.

## Results

The mean chase duration by snowmobiles was ~ 2 min (range 5 s–13 min 19 s), while the mean time between the reindeer being caught in the net and first blood sample taken was 5 min (range 1 min 20 s–35 min 16 s). The mean time taken between the first and last blood sample (immediately before release), was 20 min (range 2 min 46 s–78 min) for non-surgical procedures, and 53 min (range 38–76 min) for surgical procedures. We found no correlation between the chase time and total captures, either across years or within a year, indicating that individuals did not become more difficult to catch with increasing captures. Sample sizes for capture events in the different data sets are provided in Table S1 in Supplementary Material.

### Effect of stressor intensity and frequency on the acute stress response (P1)

We measured plasma concentrations of metanephrines, cortisol and related metabolites, as well as rectal temperatures, in winter 2018 and 2019 during 280 capture events (n) for 77 individuals (N_id_). Mean metanephrine values at first and second sampling were 5.2 nmol/L (range 1.2–20.0) and 5.1 nmol/L (range 0.8–24.0), cortisol 49.2 nmol/L (range 8.0–196.0) and 112.4 nmol/L (range 20–266), and rectal temperatures 39.1 °C (range 37.2–41.5) and 39.6 °C (range 37.7–41.4). A complete overview of the other results is provided in Table S2.

**Figure 2:**
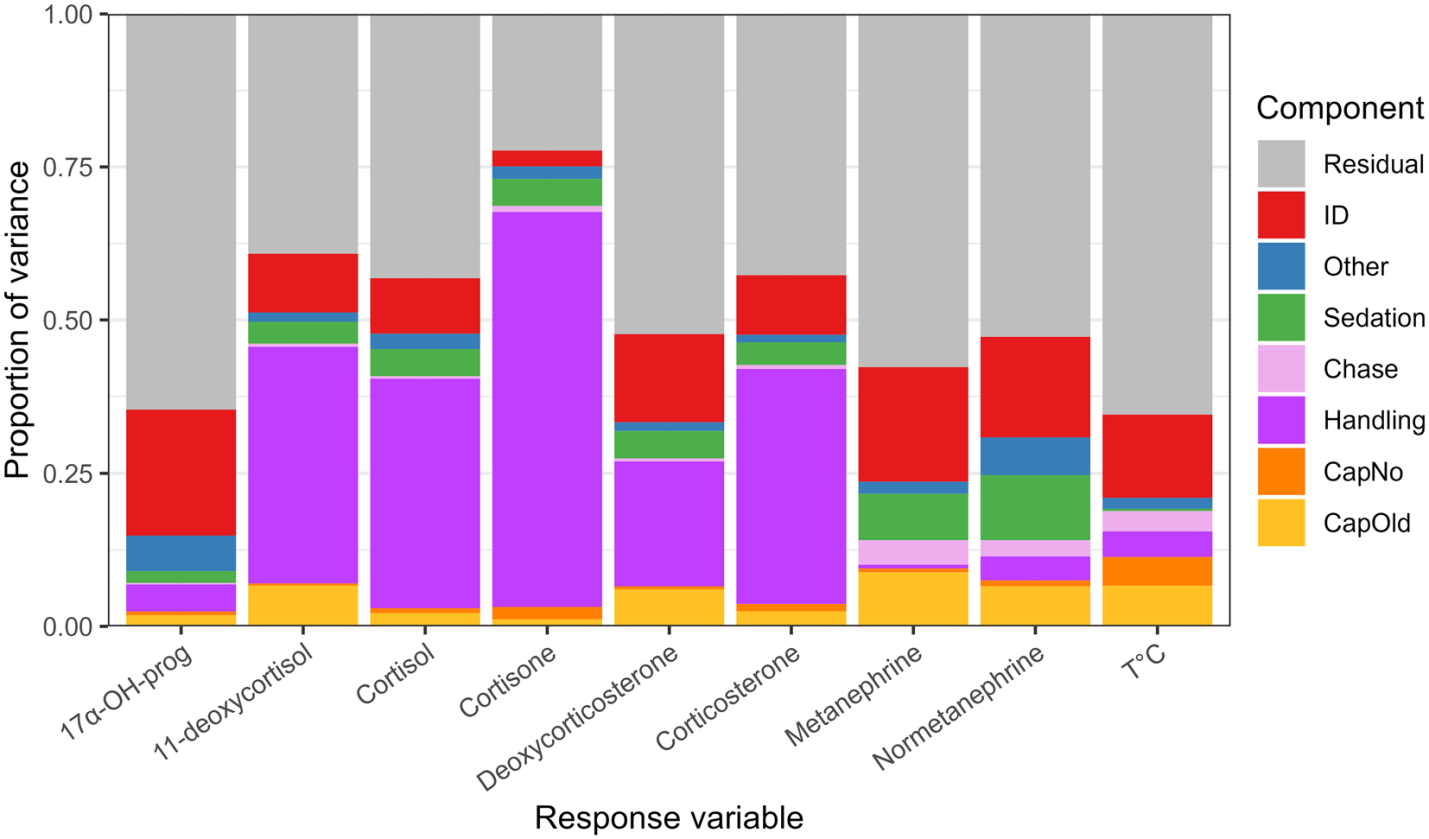
Proportion of the variance in each measured variable describing the acute stress response during capture in female Svalbard reindeer. The individual hormones and rectal temperature are explained by different groups of explanatory variables as measured with a hierarchical multivariate model. ‘ID’ refers to the repeatable individual component (i.e.: individual difference in baseline levels). Explanatory variables were grouped into other variables (blue; year and age), sedation (green), chase duration (pink), handling duration (purple), and number of captures in the current and past years (‘CapNo’, light orange, and ‘CapOld’, dark orange, respectively).

**Figure 3:**
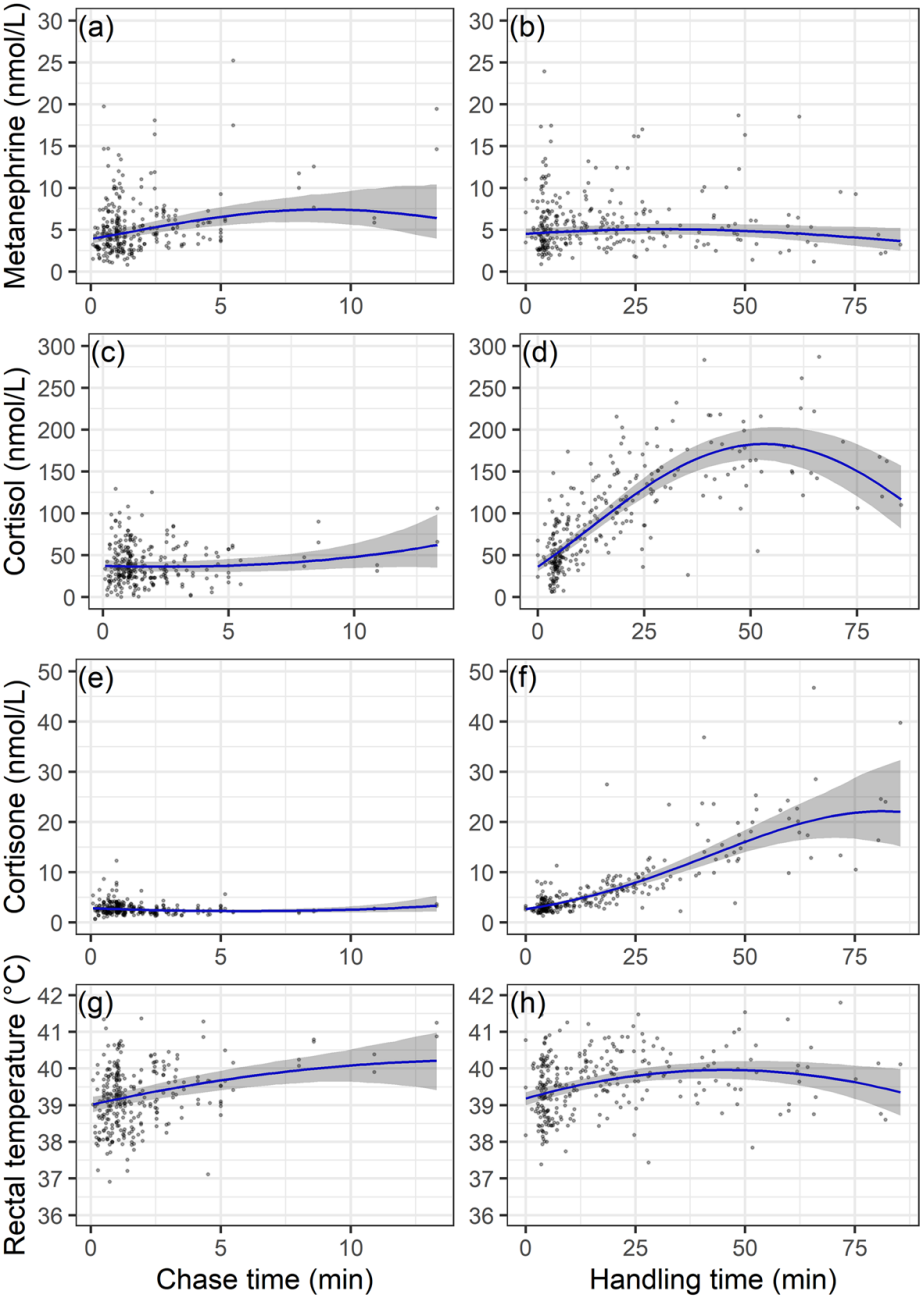
Values of the acute stress response shown as a function of stressor intensity (chase and handling duration) of female Svalbard reindeer. Chase is defined as the time from when the reindeer begins to run until it is caught in the net. Handling is defined as the time from the reindeer is caught until the blood sample was taken. Blue lines show the predicted response (with 95% credible intervals) for an average individual (6 years old, 4.5 catches in previous years, second catch of the current year, not sedated). For handling times, points are adjusted for sedation, individual, age, year of study and chase time. For chase time, points are adjusted for the handling time, i.e.: from the animal was caught until the sample was taken.

**Figure 4:**
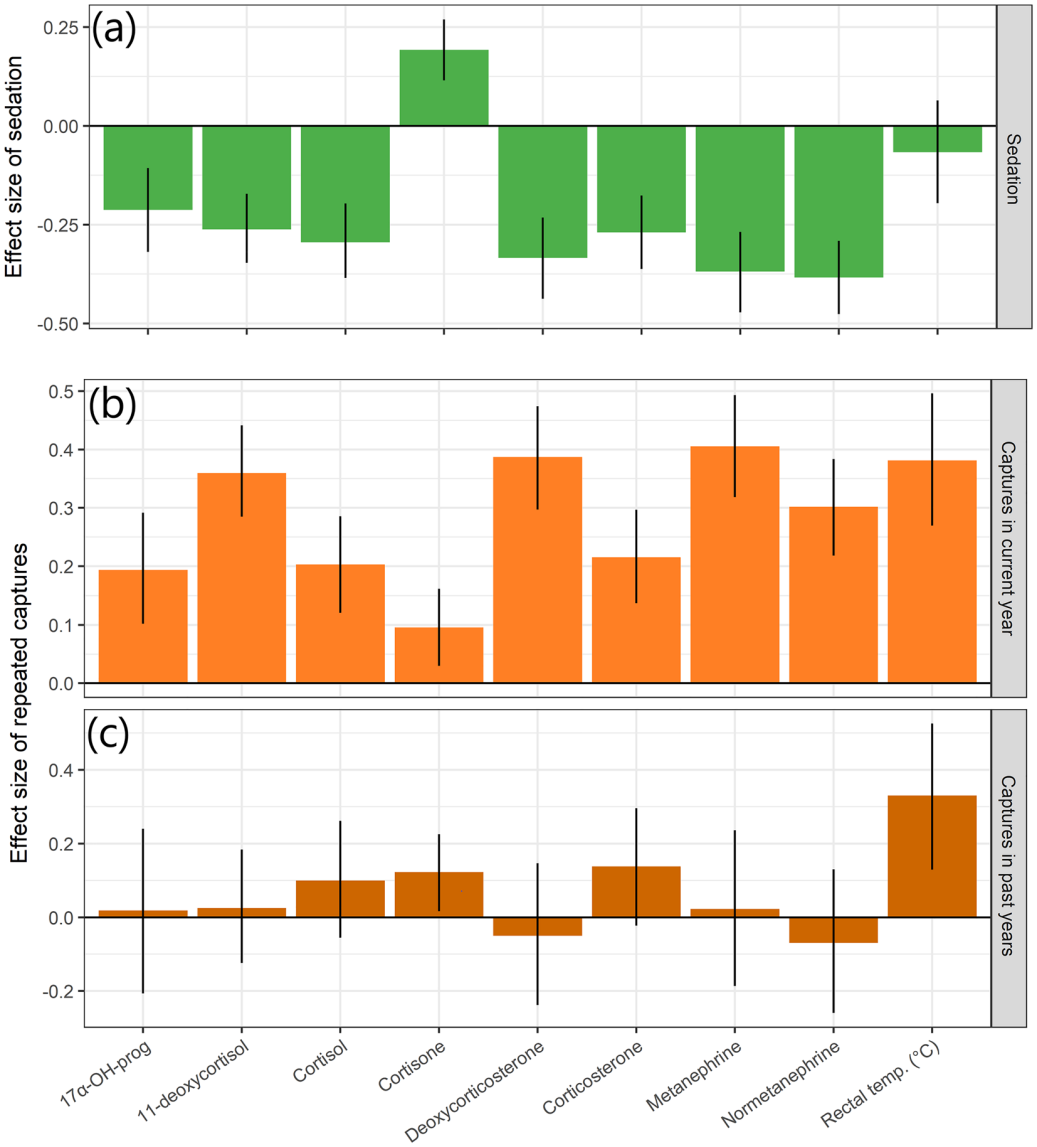
Scaled effect sizes of sedation during handling (**a**), number of captures in current year (**b**) and number of captures in past years (**c**), on each measured variable describing the acute stress response during capture in female Svalbard reindeer. A bar above zero indicates an increased stress response with increasing capture frequency, while a bar below zero indicate a reduced response. The 95% credible intervals are shown as vertical lines; non-significant results are indicated by lines crossing zero.

We evaluated the effects of captures on the acute stress response using a multivariate regression model including stress intensity (chase and handling duration, and sedation), and stress frequency (number of captures in a given and past years) while controlling for year of sampling and age of individuals. On average, this model explained 54.5% of the variance in the acute stress responses (Fig. 2; Table S3). In line with our prediction P1.1, the acute stress response increased with stressor intensity. Chase duration had a significant effect on the metanephrines and rectal temperature, which showed a steep increase with the duration of chase (Fig. 3a and h), and only slight increase with duration of handling (Fig. 3b and h). Despite this, chase time explained only 4% and 3% of the variation in metanephrines and rectal temperature, respectively (Fig. 2, Table S3). The glucocorticoids did not increase with chase times (Figs. 3c, e and S3). The duration of handling was the most important explanatory variable for cortisol and its metabolites (mean of 34%), ranging from 4% for 17α-OH-progesterone to 65% for cortisone, while this was less important (albeit significant) for the metanephrines and rectal temperature (Figs. 2, Table S3, Fig. S4). Cortisol levels peaked after approximately 55 min of handling (Fig. 3d) while cortisone increased almost linearly with handling time (Fig. 3f). In line with our prediction (P1.2), sedation depressed levels of metanephrines and all glucocorticoid levels, except cortisone, which increased during surgery (Fig. 4a). Rectal temperature was not affected by sedation.

As predicted (P1.3), there was a significant effect of capture frequency on the acute stress response, with the number of captures within a season causing increases in all metanephrines, glucocorticoids and rectal temperature (Fig. 4b), even after correcting for the effects of chase and handling time, sedation, and age of the individual (Fig. S5). The number of captures within a season explained between 1.5% (17α-OH-progesterone) and 13% (rectal temperature) of the variance in our measures. The effects of the number of captures were additive to that of handling time (all interactions with handling time overlapped zero), implying that the effect of number of captures was present immediately after capture, when the first sample was taken. Rectal temperature and cortisone were the only variables that increased with the total number of captures in previous years (range 1–11 lifetime captures: Fig. 4c) and thus the only ‘carry-over’ effect between years.

### Effect of stressor intensity and frequency on post-capture recovery (P2)

**Figure 5:**
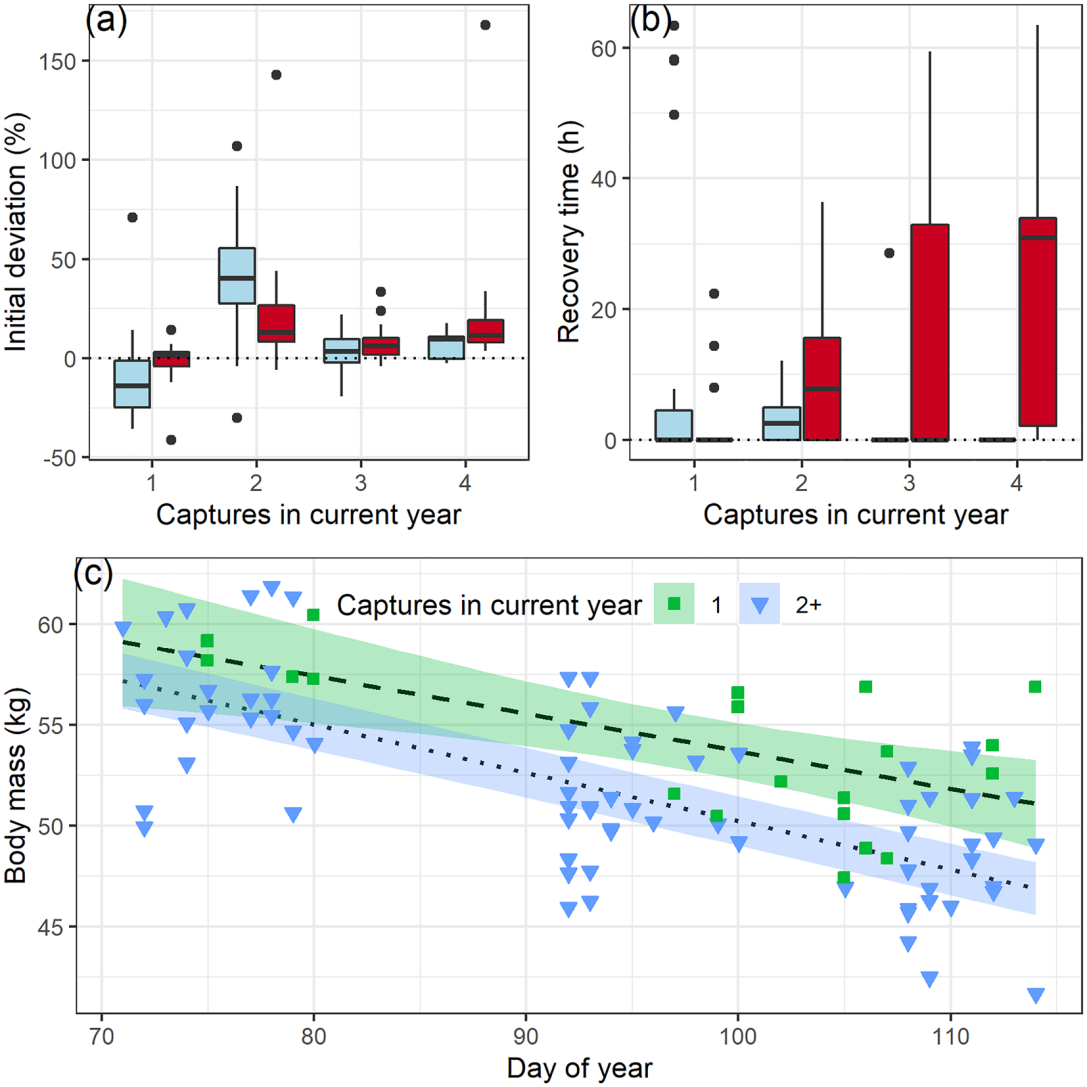
Short-term effects of repeated captures on activity and heart rate in Svalbard reindeer during winter 2018. (**a**) Initial deviation from baseline values of activity (blue) and heart rate (red) given in percentages and (**b**) time taken to return to baseline values, for activity and heart rates after each capture event. In both panels, boxes show median (solid line), the 25th and 75th percentiles (lower and upper hinges), with whiskers corresponding to max. 1.5 times the inter-quartile range. Outliers are shown as solid circles. (**c**) Winter body mass as a function of time (days since January 1st), in individuals who were captured more than once (blue, triangles) and for individuals captured only once (green, squares). Lines and shaded area show the predicted slopes with 95% confidence intervals, and points are adjusted for age and pregnancy status.

Contrary to our prediction (P2.1), relatively few individuals showed any delayed recovery in activity levels post-capture, compared with their previous baseline values, a pre-capture running mean. During the first capture event, activity levels were generally lower than baseline values by 10 ± 22%, and the mean recovery time was 11 ± 22 h, but this delayed recovery was only observed in 6 out of 23 individuals (Fig. 5a and b, Table S4). During the second capture, activity levels were up to 41 ± 33% greater than baseline values, but the mean recovery time was only 3.2 ± 3.5 h (Fig. 5a and b) and observed in 12 out of 18 individuals. The initial perturbation in activity levels (Tables S5 and S6), as well as recovery time in activity (Tables S7 and S8), was best explained by two equally parsimonious models, containing either the number of captures, or the whether the animal was subjected to surgery or not (Tables S7 and S8). The recovery time in activity decreased with each successive capture but increased by an average of 8 h if the animal was subjected to sedation. When subsetting the data for non-surgery events, the effect of capture frequency on activity was not significant.

Recovery times for HR, on the other hand, showed a marked increase with each successive capture (P2.2). Only 3 out of 21 individuals showed a delayed recovery in HR after the first capture, while all but two individuals had delayed recovery after the fourth capture. Mean recovery time after the fourth capture was ~ 24 h (range 0–63 h), with an average 25% initial deviation from baseline HR (range 4–168%). Initial perturbation values were higher than the animals’ baseline HR by ~ 20% in non-surgery events but did not deviate from baseline post-surgery (Fig. 5a). The best-fitting model for initial perturbation contained only the effect of sedation (Tables S9 and S10), while the best model for recovery times in HR contained the initial deviation (as % of baseline) and number of captures (Table S11). A greater initial deviation from baseline was associated with a longer recovery time in HR (+ 20 min per 1% deviation, Table S12), but number of captures had the greatest effect, with the recovery time increasing by 5.7 h for every repeated capture event (Fig. 5b). We included the acute stress response variables in a subset of the data, but none of these models outperformed those containing number of captures and initial deviation from baseline in activity or HR.

### Effect of stressor frequency on body mass loss, offspring survival and reproductive success (P3)

Contrary to our prediction (P3.1) repeated captures did not affect the rate of body mass loss during the field season. After accounting for age and pregnancy status, the slope of mass loss in females captured two or more times (240 g/day, SE = 53.6) was not statistically different from the estimated seasonal body mass loss estimated from cross-sectional data from animals captured only once (187 g/day; SE = 55.2; Fig. 5c, Table S13).

**Figure 6:**
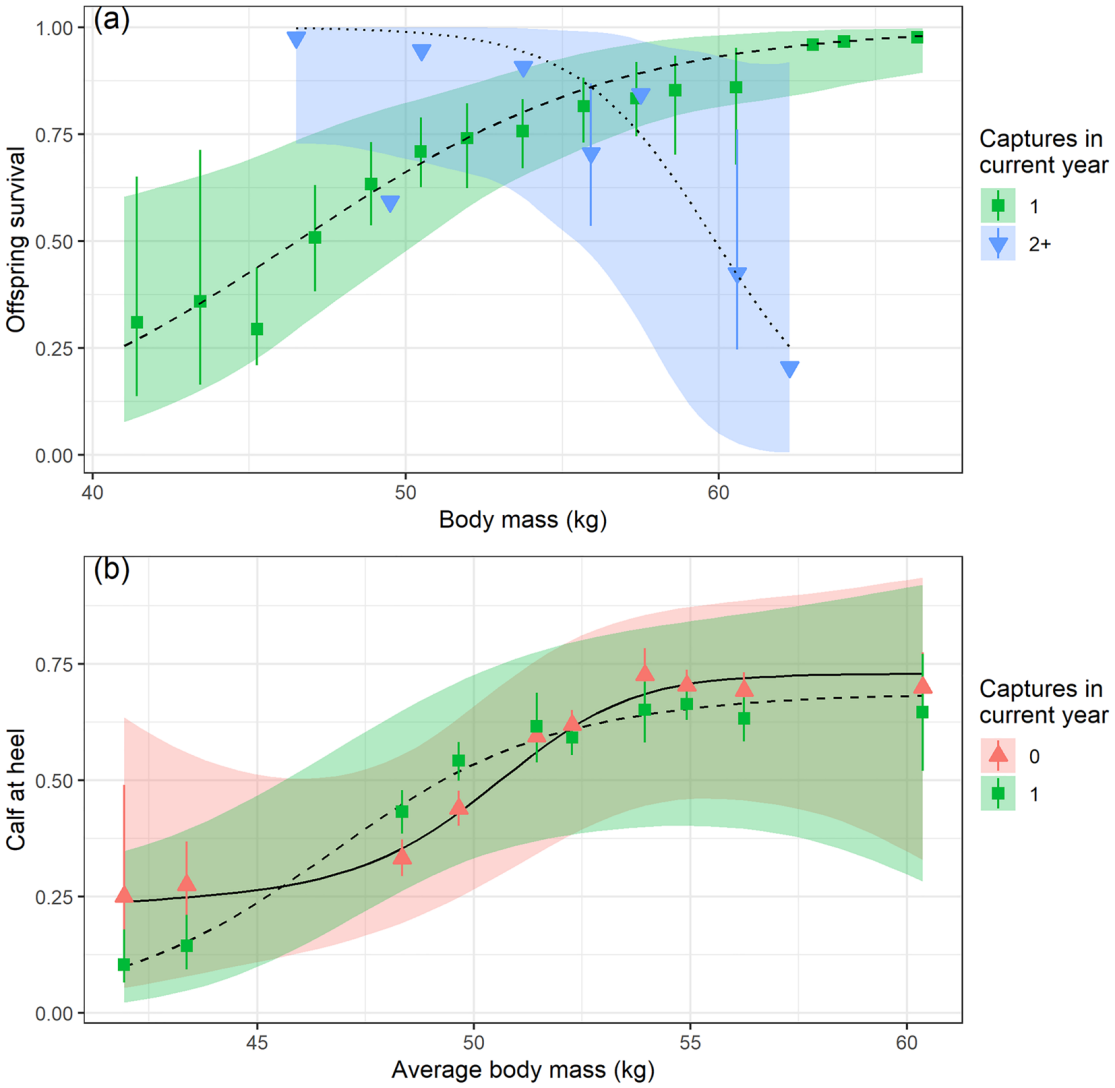
Reproductive success of female Svalbard reindeer as a function of body mass in winter (April). (**a**) Offspring survival to summer for females who were pregnant in winter and captured either once (green squares) or repeatedly (2–4 times; blue downward triangles) in winter. Each body mass relates to a specific individual. (**b**) Presence of calf at heel in females in summer as a function of the mean annual winter body mass, in females who had either not been captured (red, upward triangles, solid line), been captured once (green, squares, dashed line). Predictions are drawn from the model with lowest AICc (Tables S15 and S17). Shaded areas show 95% confidence intervals (CI) of the predictions. Points show average ± CI adjusted for year and ID, binned into equal-sized mass categories.

Nonetheless, as we predicted (P3.2) we found a significant effect of repeated captures on the probability of a female, pregnant during the winter capture, having a calf at heel (hereafter ‘offspring survival’) in August, four months later (n = 181, N_id_ = 119). The best model included an effect of mass and its interaction with the number of captures in a single year (Table S14). Individuals captured once showed the expected positive effect of body mass with offspring survival increasing from 25% to near 100% within the observed body mass range (Fig. 6a, Table S15). Surprisingly, the estimated effect of body mass on offspring survival was negative for repeatedly captured individuals, but with large uncertainties (wide confidence limits). The data do however suggest reduced offspring survival associated with repeated captures among female reindeer with large body mass. There was no significant relationship between offspring survival and timing of capture for individuals being captured only once in a year, when fitted in a model containing body mass, age and calendar day of capture (log-odds slope = 0.18, SE = 0.13, p = 0.17). We then analysed the effect of single capture events on reproductive success without the restriction of being pregnant in winter, to compare with an uncaptured ‘control’ group with unknown pregnancy status (n = 1797, N_id_ = 477). Inclusion of the interaction between capture status and body mass on reproductive success was supported by lowering ΔAICc by 3.60. With overlapping confidence intervals across all April body masses, there was no difference in reproductive success for individuals captured only once versus not captured in the current year (Fig. 6b; Tables S16 and S17).

### Effect of stressor frequency on behavioural responses (P4)

**Figure 7:**
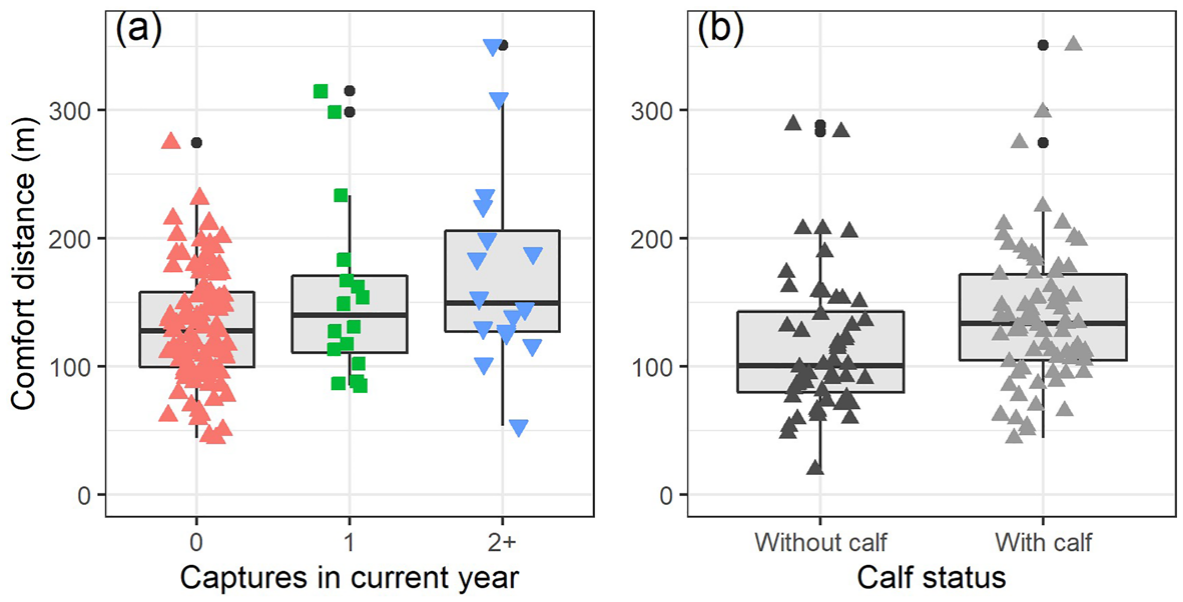
Comfort distances in female Svalbard reindeer in summer after having fled from humans approaching on foot. (**a**) Distances in individuals not captured (red triangles), captured once (green squares), or repeatedly (2–4 times; blue downward triangles) in the current year, corrected for start distance and calf status. (**b**) Distances in females with or without a calf at heel, corrected for start distance and capture frequency (predicted for zero captures). In both panels, boxes show median (solid line), the 25th and 75th percentiles (lower and upper hinges), with whiskers corresponding to max. 1.5 times the inter-quartile range. Outliers are shown as solid circles.

In the subsequent summer, approximately four months after the capture events, we investigated individual reindeer (n = 134, N_id_ = 122) responses to provocation by humans on foot. Mean alert and flight initiation distances were 135 ± 46 m and 92 ± 39 m, respectively, and, contrary to our predictions (P4), did not show a difference between individuals that had been captured or not in the preceding winter (Tables S18 and S19). Comfort distances, the distance from the approaching human at which the reindeer stopped running, was best explained by capture frequency (Fig. 7a), and whether the female had a calf at heel (Fig. 7b; Table S20). The distances averaged 100 ± 46 m for individuals who were not captured, and did not have a calf, but were ~ 42 m greater in females if captured more than once in the current year (Fig. 7a, Table S21), and an additional ~ 26 m greater with a calf.

In a subset of the data combining i) values from the acute stress response (N_id_ = 36), and ii) post-capture recovery time in HR (N_id_ = 14), we evaluated whether any responses to capture in winter could explain the behavioural response in summer. For the acute stress response, we used values from a principal component analysis grouping hormones into two main axes (Fig. S1), the first being related to the glucocorticoid response (PC1) and the other to the metanephrines (PC2). However, none of these models outcompeted the null model.

### Capture-related mortality

Between 1995 and 2018, 10 out of all 3986 (2.5‰) capture events resulted in immediate death, predominantly due to broken necks. Two adult female reindeer died (5 and 7 years old) shortly after captures in 2018. One individual died 20 days after its fourth capture, while another individual died 9 days after a single, non-surgery capture. The former lost 13 kg of body mass between first and fourth capture (37 days), resulting in a daily mass loss of 350 g/day. Both individuals were pregnant. Late winter is the main period of the year for Svalbard reindeer mortality. Although these few deaths make it impossible to arrive at a statistically robust conclusion, the high mass loss of the intensively captured individual may well have contributed to its death.

## Discussion

Our study revealed that both intensity and type of handling, as well as repeated captures within a season, can affect the physiology, behaviour, and reproductive success in Svalbard reindeer (Table 1). In general, more of the variance in measures of short-term acute stress responses was explained by intensity of capture (chase time, handling time and sedation) than the number of captures. Nonetheless, repeated captures within a field season were associated with an apparent sensitized acute stress response, as well as delayed recovery in heart rates, reduced offspring survival and an increase in comfort distances when approached by humans later in summer. These longer-term responses were not evident in reindeer that were caught only once within a season. Furthermore, we did not find a negative effect of the number of single annual capture events, even among individuals caught in up to 11 years, except for a higher rectal temperature. Our study demonstrates a distinction in the response to, and consequences of, repeated disturbance within- versus between years. Awareness of these potential impacts may be important for researchers undertaking studies with a similar degree of repeated invasiveness.

**Table 1:** Summary of responses to handling and repeated captures in female Svalbard reindeer.

Stressor	Impact	Response	Reference in paper
Chase duration	Acute stress response	Elevated serum concentrations of catecholamine (measured via metanephrines) and body temperature No change in glucocorticoids	Figure 2 Figure 3a + g Figure 3c + e Fig. S3
Handling duration	HPA axis activation	Elevated serum concentrations of glucocorticoids with increasing handling duration Small elevation in body temperature and metanephrines	Figure 2 Figure 3d + f Fig. S4 Figure 3b + h
Surgical procedure with sedation	HPA axis activation	Lower serum concentrations of glucocorticoids in sedated vs non-sedated individuals	Figure 2 Figure 4a
	Post-capture recovery	Reduced activity post-capture	Tables S6 and S8 Table S10
		Reduced HR post-capture	
Repeated captures within a season	Acute stress response HPA axis activation	Increased levels of catecholamines and glucocorticoids	Figure 4b
	Post-capture recovery	Delays in recovery time in heart rates post-capture	Figure 5a + b
		No change in activity levels post-capture	
	Behaviour	Increased comfort distance from humans 4 months later	Figure 6a
	Reproductive success	Reduced offspring survival 4 months later in heavy individuals	Figure 7a
Single annual capture events	Acute stress response	Elevated rectal temperatures with higher number of captures in previous years	Figure 4c
	Post-capture recovery	No change on post-capture activity or HR levels	
	Behaviour	No change in behaviour towards humans	Figure 6a
	Reproductive success	No change in reproductive success	Figure 7b

In line with our prediction, capture events elicited an acute stress response and HPA axis activation in Svalbard reindeer, in accordance with current stress theory^11^. The physiological stress response was demonstrated by an initial increase in both catecholamines and rectal temperature, which were greater after longer chases, followed by a prolonged increase in corticoids, which continued to increase during handling. Our results are broadly consistent with previous findings in Svalbard reindeer^14^ and other ungulates, including free-ranging impala (*Aepyceros melampus*)^16^ and vicuña (*Vicugna vicugna*)^38^, although the specific responses to being chased and restrained may vary between species. There is, however, some debate on how well glucocorticoids reflect the severity of a stressor, and animals may still remain in a state of distress when glucocorticoid levels are low^39^. Elevated glucocorticoids can also indicate that the animal is, in fact, able to mount a normal stress response^39^. The cortisol levels measured in our animals (range 8–266 nmol/L) overlap with reported “baseline” levels for domestic reindeer (~ 30 nmol/L)^40^ and were lower than those measured during capture by helicopter in caribou (~ 300 nmol/L)^18^. Since the most important factor explaining variation in glucocorticoids was the duration of handling, it is likely that a continued activation of the HPA axis occurred while reindeer were restrained. For instance, stressor severity increases both the levels and duration of elevated serum cortisol in sheep (*Ovis aries*)^41^. Serum cortisol could also continue to rise after release in reindeer handled for shorter periods of time, but this is not something we could measure. In contrast to the other corticoids measured, cortisone increased linearly with handling time. Cortisol is converted to its inactive form, cortisone, via 11β-hydroxysteroid dehydrogenase (11-β-HSD)^12^. If all circulating cortisone above a given baseline arises from elevated cortisol levels, the rapid and continued increase in cortisone may reflect a stress response which would be undetected when measuring cortisol alone^42^. In that sense, cortisone could potentially serve as a biomarker of accumulated stress during long handling sessions and during sedation.

The surgical procedure involving sedation, another form of stressor intensity, also affected the acute stress response. In support of our predictions, serum concentrations of catecholamines and all glucocorticoids, except cortisone, were lower in sedated individuals after correcting for handling duration. This was expected as the drug used (medetomidine) is α2 adrenergic agonist which typically reduces the stress response in animals^19^. The opposite pattern has been observed in domestic reindeer (*R. t. tarandus*) and sheep subjected to medetomidine^20,43^. This difference could be due to different dosages used, or possibly species- and age- dependency^43^. Sedatives with different chemical pathways can also affect the physiological stress responses differently. For instance, subjection to azaperone (a neuroleptic drug), affected neither serum cortisol nor heart rate in partially sedated mule deer (*Odocoileus hemionus*)^17^, while heart rate, but not serum cortisol, increased in Iberian ibex (*C. pyrenaica*)^44^. Capture using chemical immobilization can also induce short-term (hours to c. 5 days) behavioural changes, such as movement rates in mule deer^45^ and free-ranging bison (*Bison bison*)^46^, and reduced activity levels in Alpine ibex^28^. In our study, post-capture activity levels and heart rates were lower after surgery, which included sedation, than regular capture, without sedation. Similar results have been found in goats (*C. hircus*)^21^ and could be explained by a lasting effect of the sedative despite using the recommended dosages for reversal^20^. Further, post-capture recovery could be prolonged due to pain which may not have been fully mitigated by the administered non-steroidal anti-inflammatory drug (meloxicam), although similar doses of meloxicam have produced assumed therapeutic concentrations for up to three days in semi-domesticated reindeer^47^. The effects of sedation are likely to depend on the drug used and the capture method^17^, as well as species’ differences^20^, emphasizing the importance of evaluating the effects of specific sedatives on the relevant species in question.

As predicted, repeated captures within a season were associated with a heightened acute stress response including elevated corticosteroids during each capture event. This response is consistent with a sensitized physiological stress response^11^ and likely reflects a lack of habituation to handling in Svalbard reindeer. Similar results have been found in free-ranging mouflon (*Ovis musimon*)^15^, whereas in impala^16^ and chital deer (*Axis axis*)^48^, both confined in enclosures, the cortisol response decreased as individuals were repeatedly captured. The difference in these responses could reflect a situation-dependent habituation.

We did not observe any changes in recovery of activity levels post-capture (excluding the effects of sedation), but most individuals in our study displayed a delayed recovery time in HR, increasing in duration with each additional capture, thus providing partial support to our predictions. After the fourth capture, some individuals spent several days (range 2–60 h) before returning to normal heart rates. This could prove to be an under-estimation since long recovery times may become confounded with the seasonal change in (increasing baseline) heart rate. We did not find a correlation between recovery times and catecholamines or glucocorticoids levels, possibly because elevated glucocorticoids during an acute stress response do not necessarily reflect a propensity for chronic/delayed stress responses^39^, or simply because of our small sample sizes. Nonetheless, an elevated HR could reflect a greater energy expenditure^49^ post-capture, which if not compensated by higher foraging activity, could potentially increase the rate at which body reserves are depleted.

Several studies have found negative effects of capture on body mass in small mammals, which are sensitive to lost foraging time due to relatively high metabolic demands^50,51^. Furthermore, in some larger mammals, which are typically less sensitive to short interruptions in foraging patterns, such as grizzly and American black bears^8^, and Eurasian beavers^9^, repeated captures have still resulted in lifelong depressed body mass (although this was not the case in polar bears^22^ or male Eurasian brown bears^52^). While we found no statistically significant effect of repeated captures on body mass, a trend in 20% greater mass loss (50 g/day) for individuals caught multiple times could be of concern as maternal late winter body mass is a dominant factor for reproductive success in Svalbard reindeer^53^. The significant interaction between repeated captures and body mass on offspring survival indicate that repeated captures reduced offspring survival in heavier females (but not in lighter females), which otherwise would be likely to reproduce successfully^37^. Although 2018 was a year with relatively benign winter conditions^54^, a similar capture regime during a harsh winter might have resulted in more individuals reaching the lower body mass threshold for successful reproduction, and potentially compromise their own survival. Although we only observed one mortality in the repeatedly captured group, the high mass loss of this intensively captured individual (350 g/day), may have contributed to its death.

We found no effect of single capture events on the reproductive success in Svalbard reindeer (when compared with uncaptured individuals), nor any evidence for reduced reproductive success with increasing number of lifetime captures, in line with both our previous analyses^14^, and studies of caribou (*R. tarandus*) and alpine ibex (see^28^ and references therein). However, single capture events involving sedation/immobilization have been associated with reduced calf survival in some ungulates, including moose (*Alces alces*)^55^ and mountain goats (*Oreamnos americanus*)^26^. These apparent differences might depend on the timing of capture relative to the reproductive cycle. For instance, capture of mothers with young offspring may increase the risk of abandonment^26^, while captures, and especially chemical immobilization, in the late gestational stages may increase the risk of damage to the foetus^23^. Although the last capture events occurred less than two months prior to parturition, we found no relationship between timing of captures (range of 55 days) and offspring survival. Further, repeated captures were more important in explaining offspring survival than the surgical treatment involving sedation, suggesting that the timing of captures during this gestational period does not pose a great risk for offspring survival in Svalbard reindeer.

The sensitization of the captured individuals was to a small extent detectable four months later in our provocation experiment. Contrary to our prediction, only comfort distances, which is the distance the reindeer created between the disturbance (approaching human) and itself, were greater in individuals that had been repeatedly captured in late winter. Therefore, it seems possible that repeated negative experiences with snowmobiles in late winter can elicit a greater flight response when approached by humans walking on foot, several months later. Yet, the distances between the groups differed by less than 50 m, distances, which are unlikely to affect foraging activity or increase energy expenditure given the low levels of exposure to humans throughout the year^56^.

There are many research questions of importance for conservation, management, and basic research, that cannot be answered without repeated captures. For example, our own study of the daily energy expenditure in wild Svalbard reindeer, which enhanced our understanding of energy balance during the food-depleted winter in this high-Arctic species experiencing rapid climate change^3,57^. Overall, this capture regime appears to have elicited a sensitized, short-term stress (hours to days) responses but with minor long-term consequences. Yet, we observed reduced offspring survival in a handful of individuals captured repeatedly in the same year. In our case, few individuals relative to the populations size were needed (ca 20 out of the female population of > 2200 individuals), thereby reducing the potential for population-level consequences, but arguably not for the individual animal’s welfare. In many countries legislation systems ensure that studies meet guidelines for weighing the potential trade-offs between animal welfare and knowledge acquisition, so that research practises are in line with animal welfare policies and that animal research is based on sound science. Transparency in reporting the consequences of research practises is essential to inform these ethical debates.

Lastly, our study contributes to the growing body of literature showing that reported acute stress responses, post-capture recovery rates and long-term fitness consequences of capture events vary between species and contexts^5,10^. We emphasize the importance of researchers evaluating the effects of capture and handling protocols on their study species, as even closely related species may display large differences in their response to capture and handling.

## Methods

### Study system and animals

The study was carried out in Colesdalen and Reindalen of Nordenskiöld Land, Svalbard (77°50’–78°20’N, 15°00’–15°60’E)^58^. The population size was estimated at c. 2200 individuals in 2018^58^, with c. 400 (18%) marked with individually identifiable tags as part of a long-term capture-mark-recapture study^37^. Hunting takes place between 15 August and 20 September and removes max. 7% of the total population size^59^. Outside the hunting season, human presence during the snow-free period is rare. Typically, the ground is snow covered from October through mid-June^60^. Svalbard reindeer give birth to a single calf in early to mid-June^53^.

### Capture and handling protocol (winter)

Captures occurred annually in March–April (hereafter “winter”) (Fig. 1a). Two snowmobile drivers identified an individual from several hundred metres, and slowly steered it in a desired direction. When the reindeer was less than 100 m away, or if it began to run, a 7 m × 4 m net was stretched between the snowmobiles. The two snowmobiles passed either side of the reindeer dropping the net over it, which entangled it so that it fell in the snow. Caught reindeer were manually restrained, and legs tied together with a rope. A video is available online (https://youtu.be/rFBx_HqmFn0). Once restrained, jugular blood samples and rectal temperatures were taken, the reindeer were weighed, and pregnancy status and back fat depth were measured using an ultrasound machine.

During the 2018 winter field season, a subset (N_id_ = 21) of the marked population was captured up to four times between 15 March and 25 April^3^, while other marked reindeer were captured only once during the same period (N_id_ = 89). During the first capture biologgers (DST Centi-HRT, StarOddi, Iceland) were implanted subcutaneously in the chest region. Sedation was induced by medetomidine (Domitor vet, Orion Pharma Animal Health, Finland; dose ~ 0.14 mg kg^−1^ body mass, BM) or dexmedetomidine (Dexdomitor vet, Orion Pharma Animal Health, dose ~ 0.07 mg kg^−1^ BM) and reversed by atipamezole (Antisedan 5 mg mL^−1^, Orion Pharma Animal Health; 5 mg mg^−1^ medetomidine or 10 mg mg^−1^ dexmedetomidine) once the surgery was completed. The animals were blindfolded while sedated. Local anaesthetics (bupivacaine, 2.5–5 mg Marcaine, AstraZeneca) was administered at the surgical site, and analgesic NSAID (0.5 mg kg^−1^ meloxicam; Metacam, Boehringer Ingelheim Vetmedica GmBH) was administered intramuscularly (further details in^57^). About three weeks later, these reindeer were captured up to three additional times, with the second and third capture being 2–5 days apart and the fourth capture occurring 10–17 days later. All procedures performed in this study were in accordance with relevant Norwegian regulations and guidelines. The capture and live animal handling procedures were approved by the Norwegian Food Safety Authority (permit no. 17/237024) and the Governor of Svalbard (permit no. 16/01632-9). Methods are reported following ARRIVE guidelines.

### Biologger data handling

The heart rate (HR) biologgers were recovered after 12 months. HR was recorded every 15 min, alongside an index for signal quality which facilitated a filtering process for retaining more reliable data^57^. GPS-collars (Vectronic Aerospace GmbH, Germany), containing an activity sensor recording gravitational acceleration along two axes, backward-forward and right-left movements were also fitted. An internal algorithm calculates mean acceleration in each axis every 5 min, resulting in a value between 0 and 255. We used the sum of activity in both axes as an index of overall activity levels.

### Hormone assays

In 2018 and 2019, blood samples were collected twice, at the beginning and end of handling (n = 280, N_id_ = 77). Blood was collected into serum vacutainers and kept unfrozen until returning from the field each day (1–8 h). After centrifuging serum was kept frozen at − 20 °C until further analyses. Metanephrines (normetanephrine and metanephrine) and cortisol with related metabolites (cortisone, corticosterone, 11-deoxycortisol [11-DEO], deoxycorticosterone [DOC] and 17α-hydroxy progesterone [17-OH-prog]) were analysed by liquid chromatography tandem mass spectrometry (LC–MS/MS) at the Hormone Laboratory, Oslo University Hospital, Norway^61^ accredited according to NS-EN ISO/IEC 17,025:2017 for hormonal measurements in humans. Quantification limits were 0.2 nmol/L (normetanephrine), 0.1 nmol/L (metanephrine), 0.5 nmol/L (cortisol), 0.2 nmol/L (corticosterone), 0.2 nmol/L (11-DEO), 0.71 nmol/L (DOC) and 0.2 nmol/L (17-OH-prog). The analytical CV% ranged from 6 to 15%, and the accuracy ranged between 90 and 110% for all steroid hormones. Individual samples taken on the same day (1st and 2nd sample) were analysed together.

### Census and provocation study (summer)

Annual surveys in early August, assessed the presence/absence of calves associated with marked females. Average group size in summer is 2–3 individuals^62^, facilitating assignment of mother–calf pairs. In the open landscapes reindeer can be identified from long distances (> 1 km) with binoculars and telescopes. In 2018 and 2019, we performed a provocation study to investigate potential effects of winter capture on reindeer flight behaviour in summer^63^ to the approach of a human on foot using both marked and unmarked adult females (≥ 2 years old, n = 134, N_id_ = 122). Specific details are given in the Supplementary Material: Sect. 1.1. The following terminology was used, modified from^63^:

- Encounter distance: Distance between the observer and the reindeer when approached.
- Alert distance: Distance when the reindeer was standing, head above the horizontal, clearly attentive, staring at the observers, or starts to walk away.
- Flight initiation distance: Distance from the approaching observer when the reindeer initially took flight.
- Comfort distance: Distance between the observer and the reindeer when it stopped running and/or began grazing.

### Statistical analyses

All statistical analyses were performed in R version 4.1.1^64^. For all model selections described below, candidate models were compared using Akaike’s Information Criteria for small sample sizes (AICc)^65^. The models with the lowest AICc were considered as the best models. In case of several competing models, ΔAICc < 2, the one with fewest explanatory variables was selected.

#### Physiological stress response

We first used a standard principal component analyses approach to explore correlation structure between all measured hormones and metabolites (Fig. S1). Since these variables were strongly correlated (Fig. S2), we used a hierarchical multivariate model (HMSC)^66,67^, a multivariate mixed effect regression controlling for individual heterogeneity, to jointly investigate the drivers of variation in hormone levels. This allowed us to test the effects of the capture regime, while accounting for both the interconnectedness of the stress hormones, and the non-independence of samples from the same individual. Cortisol and corticosterone were square root-transformed, while other hormones were log-transformed prior to analysis to meet the assumptions of a constant variance and linear relationships^68^. Rectal temperature, also an indicator of physiological stress, was not transformed. All variables were then scaled to a mean of 0 and a standard deviation of 1 to facilitate convergence and make comparison of effect sizes easier. We quantified the effect of different stressors: sampling variables (was the reindeer sedated prior to blood sample), duration of the stressor (chase time: time taken from beginning of the chase to capture), handling time (time between capture and sample) and their quadratic effects, and two measures of stressor frequency (both number of captures this year and total number of captures in previous years). We controlled for potential differences between the two years in our study by adding year as a factor in our model. Because body condition declines non-linearly with age, we fitted age as a quadratic effect. Finally, we fitted an individual intercept as a random effect to account for individual heterogeneity. No interactions were considered. Models were run for 130,000 iterations, including 30,000 iterations of burn-in with a thinning of 100 with default priors for gaussian distribution. The significance of effects was determined based on their 95% credible intervals (CrI). R^2^ was assessed using the function “R-squared” of the “HMSC” package. We estimated the explained variation in the model due to random and fixed effects using the function “variPart” of the “HMSC” package. We grouped the variance explained by age, age squared and year into a single value (hereafter “other”), to simplify presentation of the results.

#### Post-capture recovery

We quantified post-capture recovery time as an estimate of the time taken (in hours) for values of heart rate and activity to no longer be substantially different from predicted baselines (see supplementary material Sect. 1.2). In a subsequent step, we investigated these recovery times using linear mixed effects regressions to account for non-independence of repeated measures from each individual^69^. We used the following candidate explanatory variables: body mass, pregnancy status (factor), age (numeric), number of captures that year (grouped by 1 capture vs. 2 +), total lifetime captures, initial deviation from baselines (as % of baseline), and rectal temperature during handling. We also included models with the effect of sedation, instead of number of captures. Individual identity was fitted as a random effect.

We also investigated whether captures affected the rate of body mass loss during the field season. Here, we compared body mass in adult females (ages 4–8) captured only once (N_id_ = 21) and captured more than once (N_id_ = 30) in 2018. We used a linear mixed-effects model including age as numeric variable, and reproductive status as a factor, and individual as a random effects term. We fitted body mass as a function of day of the year as an interaction with number of captures that year (1 capture vs. 2 +), to compare the seasonal mass loss trend (population estimate from single captures) against the mass loss in repeatedly captured individuals.

#### Offspring survival and reproductive success

We fitted models with the presence of a calf in summer (“calf at heel”) as a binomial response variable, using only females for whom pregnancy had been confirmed in winter the same year, hereafter “offspring survival”. We used a generalized linear mixed model, assuming binomial error and a logit link function, with individual and year as random effects. We then tested models including combinations of winter body mass and times captured (grouped by 1 capture vs. 2 +) as either additive predictor variables or with an interaction, and only years when repeated captures of individuals occurred were used (2016–2021, n = 181, N_id_ = 119). We compared models with a variable indicating whether animals had been subjected to surgery or not, with those containing number of captures, as both variables could not be used in the same model. We also assessed the potential effect of timing of capture in relation to gestation. In this model, all data including individuals only captured once per year was used, for all available years of data (1995–2021, n = 611, N_id_ = 300), and the day of the year (DOY) was included as an explanatory variable. Next, we fitted models with calf-at-heel as the response variable (hereafter ‘reproductive success’), for all marked females in the population, including those who had not been captured in the winter the same year (hence unknown pregnancy status), for all available years of data (1995–2021, n = 1861, N_id_ = 488). We used a generalized linear model, assuming binomial error and a logit link function, using the combinations of average winter body mass and times captured (grouped by 0 or 1) as either additive or interacting effects. We fitted year, either as a factor or as a random intercept, and ID as random intercept. In all of the above models, body mass was scaled to a mean of 0 and standard deviation equal to 1.

#### Provocation by humans

The determinants of alert, flight, and comfort distances were fitted with linear regressions with the following candidate explanatory variables: Encounter distance, calf status (factor), times captured the previous winter (grouped 1 vs. 2 +) and year (2018/2019). Age was not included because it could not be determined for unmarked individuals, but only responses by adult females (> 2 yrs) were included. We also tested for relationships between variables measured in winter (hormones and post-capture recovery) in a subset of the data. We included log-transformed encounter distances in each model as a control variable.

## Supplementary Information

Supplementary Information.

## Data Availability

The data used in this article is available on Figshare through the following link: 10.6084/m9.figshare.21119023.
