# Author Correction: Stress responses to repeated captures in a wild ungulate

**DOI:** 10.1038/s41598-023-29744-0

**Published:** 2023-02-15

**Authors:** L. Monica Trondrud, Cassandra Ugland, Erik Ropstad, Leif Egil Loe, Steve Albon, Audun Stien, Alina L. Evans, Per Medbøe Thorsby, Vebjørn Veiberg, R. Justin Irvine, Gabriel Pigeon

**Affiliations:** 1 Faculty of Environmental Sciences and Natural Resource Management, Norwegian University of Life Sciences, NO-1432 Ås, Norway; 2 Centre for Biodiversity Dynamics, Institute of Biology, NTNU, NO-7491 Trondheim, Norway; 3 Faculty of Veterinary Science, University of Life Sciences, NO-1432 Ås, Norway; 4 The James Hutton Institute, Craigiebuckler, Aberdeen, AB15 8QH UK; 5 Department of Arctic and Marine Biology, The Arctic University of Norway, NO-9037 Tromsø, Norway; 6 Department of Forestry and Wildlife Management, Inland Norway University of Applied Sciences, Campus Evenstad, NO-2406 Elverum, Norway; 7 Hormone Laboratory, Department of Medical Biochemistry and Biochemical Endocrinology and Metabolism Research Group, Oslo University Hospital, Aker, Oslo, Norway; 8 Department of Terrestrial Ecology, Norwegian Institute for Nature Research, Torgarden, P.O. Box 5685, NO-7485 Trondheim, Norway; 9 Frankfurt Zoological Society, South Africa Street, P.O Box 100003, Addis Ababa, Ethiopia; 10 Institut de Recherche Sur Les Forêts, Université du Québec en Abitibi-Témiscamingue, Rouyn-Noranda, QC J9X 5E4 Canada

Correction to: *Scientific Reports* 10.1038/s41598-022-20270-z, published online 29 September 2022

In the original version of this Article we used Ref 38 to support the following statements:

"Our results are broadly consistent with previous findings in Svalbard reindeer [14] and other ungulates, including free-ranging impala (*Aepyceros melampus*) [16], guanacos (*Lama guanacoe*) [38], and vicuña (*Vicugna vicugna*) [39], although the specific responses to being chased and restrained may vary between species. For instance, in guanacos, cortisol increased with handling time, but corticosterone did not [38]. In contrast, both cortisol and corticosterone increased during handling in our study. There is, however, some debate on how well glucocorticoids reflect the severity of a stressor, and animals may still remain in a state of distress when glucocorticoid levels are low [40]. Elevated glucocorticoids can also indicate that the animal is, in fact, able to mount a normal stress response [40].”

After publication it has come to our attention that Reference 38 was retracted in 2015 [1]. We therefore do not believe it is appropriate to use it to support these statements. As a result, these sentences are revised and now reads:

"Our results are broadly consistent with previous findings in Svalbard reindeer [14] and other ungulates, including free-ranging impala (*Aepyceros melampus*) [16], and vicuña (*Vicugna vicugna*) [39], although the specific responses to being chased and restrained may vary between species. There is, however, some debate on how well glucocorticoids reflect the severity of a stressor, and animals may still remain in a state of distress when glucocorticoid levels are low [40]. Elevated glucocorticoids can also indicate that the animal is, in fact, able to mount a normal stress response [40].”

Reference:

1. Retraction: “Do cortisol and corticosterone play the same role in coping with stressors? Measuring glucocorticoid serum in free-ranging guanacos (*Lama guanicoe*)”, by R. Ovejero, A. Novillo, M. Soto-Gamboa, M.E. Mosca-Torres, P. Cuello, P. Gregório, G. Jahn and P. Carmanchahi. J. Exp. Zool. (2015). https://doi.org/10.1002/jez.1918

