# Supplementary material for: Stress responses to repeated captures in a wild ungulate
Source: Sci Rep. 2022 Sep 29;12:16289. doi: 10.1038/s41598-022-20270-z (PMC9522855; doi:10.1038/s41598-022-20270-z)
Supplement: Supplementary file 1 — Supplementary Information. [file 41598_2022_20270_MOESM1_ESM.pdf]

Supplementary material for "Stress responses to repeated captures a wild ungulate"

Trondrud, L. Monica[*a], Cassandra Ugland[a], Erik Ropstad, Leif Egil Loe, Steve Albon, Audun Stien, Alina L. Evans, Per Medbøe Thorsby, Vebjørn Veiberg, R. Justin Irvine, Gabriel Pigeon

*Corresponding author: monica.trondrud@nmbu.no, monica.trondrud@gmail.com

[a]L. Monica Trondrud and Cassandra Ugland are joint first authors.

Contents

List of figures 2

List of tables 2

1 Supplementary methods 3

1.1 Summer census and provocation study 3

1.2 Statistical analyses – Post-capture recovery times 3

2 References 3

3 Supplementary figures 5

4 Supplementary tables 10

List of figures

Figure S1. Principal components of the acute stress response....5
Figure S2. Correlation between the measured variables relating to the acute stress response. .5
Figure S3. Values of the acute stress response as a function of chase time. 6
Figure S4. Values of the acute stress response as a function of handling time. 7
Figure S5. Values of the acute stress response as a function of number of captures t 8
Figure S6. Example of return to baseline in activity. 9

List of tables

Table S1. Summary of sample sizes 10
Table S2. Summary of the physiological stress parameters 11
Table S3. Variance explained for each physioloigcal stress parameter. 12
Table S4. Recovery times and initial deviations for activity (ACT) and heart rate (HR). 13
Table S5. AICc model selection with initial deviation in activity levels....14
Table S6. Summary best model explaining initial deviation in activity levels....15
Table S7. AICc model selection with recovery time of activity levels. 16
Table S8. Summary of best model explaining recovery time activity levels....17
Table S9. AICc model selection with initial deviation in heart rates.. 18
Table S10. Summary of best model explaining initial deviation in activity levels. 19
Table S11. AICc model selection with recovery time of heart rates 20
Table S12. Summary of best model explaining recovery time heart rates 21
Table S13. Summary of model explanining body mass change in winter....22
Table S14. AICc model selection with offspring survival....23
Table S15. Summary of best model offspring survival 24
Table S16. AICc model selection with reproductive success 25
Table S17. Summary of best model of reproductive success 26
Table S18. AICc model selection with alert distances 27
Table S19. AICc model selection with comfort distances....28
Table S20. AICc model selection with comfort distances....29
Table S21. Summary of best model of comfort distances....30

1 Supplementary methods

1.1 Summer census and provocation study

Two persons dressed in dark clothing (the observers) encountered reindeer by directly approaching them and measuring the distances between the reindeer and the observers using Leica Geovid 7x42 BDA laser binoculars (1 m accuracy at 1000 m). Before each encounter, the observers registered the wind direction (towards or away from animal or group) and calf status of females. The observers then approached the reindeer by walking directly towards the animal or group in an uninterrupted pattern, stopping only to measure distances at which the reindeer altered their behaviour. All distances were measured between the observer and the closest animal in the group. In order to complete a provocation, the observers had to be visible to the reindeer from the start of the provocation until the reindeer initiated their flight. There were no significant differences in alert, flight initiation or comfort distances when the wind was towards or away from the reindeer, and due to some missing information on wind direction, this variable was excluded from the candidate models to improve sample size.

1.2 Statistical analyses – Post-capture recovery times

Because reindeer exhibit both activity bouts, daily cycles and seasonal changes in activity and heart rate[1,2], we first needed to identify deviations from normal patterns of activity and heartrate. For each individuals, we first calculated moving averages for activity and heart rate over 9-hour window at 1-hour repeats (based on visual inspection of the bouts) to remove any effect of activity/foraging bouts on the baseline estimates. Second, to account for the non-linear change in activity and heart rate due the end of winter, these moving averages were then fitted in generalized additive models (GAM) against time (date and time of day) using a thin plate regression spline[3], and an autoregressive function (AR1) was used to account for temporal autocorrelation. We included activity level as an explanatory variable in the

estimates for heart rate baselines to account for any effect of activity-induced elevations in heart rate. The predictions from these GAMs were used to quantify a baseline value with standard error (SE) for each response variable per individual over the entire period. Third, we quantified the return to baseline after each captures. Because time and time since last capture are inherently correlated, it was impossible to use both in a single additive model. By using the residuals of a first gam we assume that most of the long-term temporal trend are seasonal and that important deviations from this trend represent perturbations. Therefore, the residuals of each model (the deviations from the expected baseline) from moment of capture until up to 14 days after capture (or the next capture if sooner), were fitted in new GAMs for each individual and for each capture event, with time since capture as explanatory variable. A gam was used since the return to baseline is expected to be non-linear; with deviation being initially high, then declining and stabilising around zero (Figure S6). The recovery time was calculated as the difference between time of capture, and the first timestamp at which the standard error of around the residual regression and baseline regression overlapped.

2 References

1. Arnold, W. *et al.* Circadian rhythmicity persists through the Polar night and midnight sun in Svalbard reindeer. *Sci. Rep.* **8**, 14466 (2018).
2. Trondrud, L. M. *et al.* Determinants of heart rate in Svalbard reindeer reveal mechanisms of seasonal energy management. *Philos. Trans. R. Soc. B Biol. Sci.* **376**, 20200215 (2021).
3. Wood, S. N. *Generalized Additive Models: An Introduction with R*. (Chapman and Hall/CRC, 2017).
4. Johnson, N. L., Kotz, S. & Balakrishnan, N. *Continuous Univariate Distributions: Volume 2*. (1995).

3 Supplementary figures

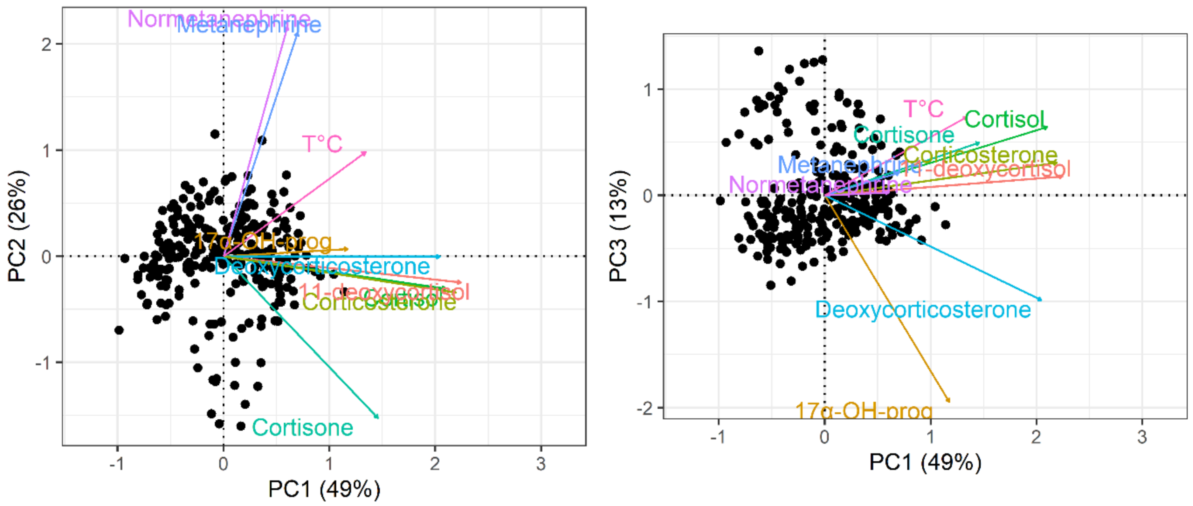

Figure S1. Relationships between the extracted components from a principal component analyses of the measured variables relating to the acute stress response in female Svalbard reindeer during captures.

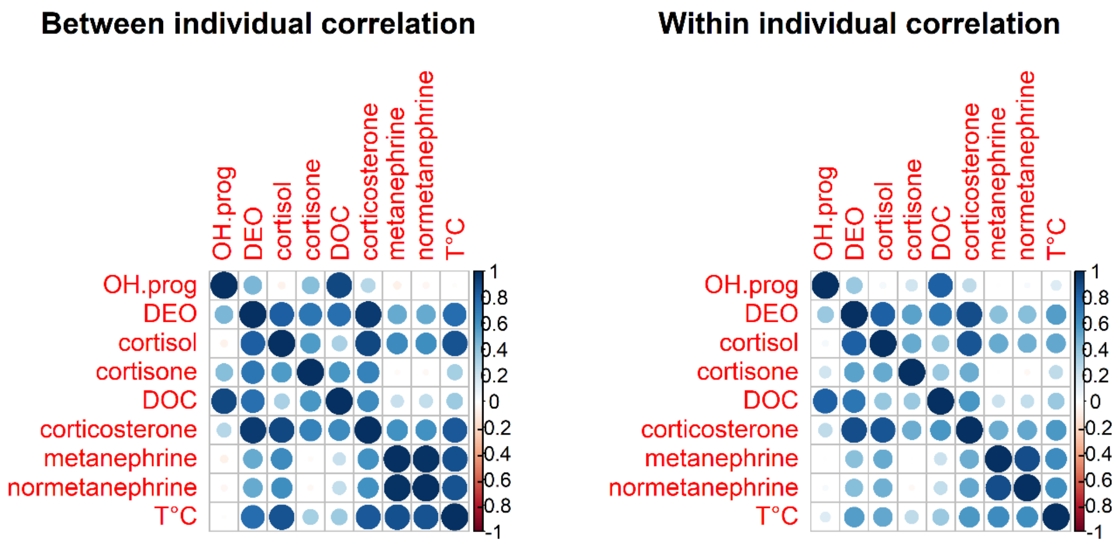

Figure S2. Between (left panel) and within (right panel) individual correlation between the measured variables relating to the acute stress response in female Svalbard reindeer during captures. Abbreviations: OH.prog = 17α-OH-progesterone, DEO = 11-deoxycortisol, DOC = deoxycorticosterone, T°C = rectal temperature.

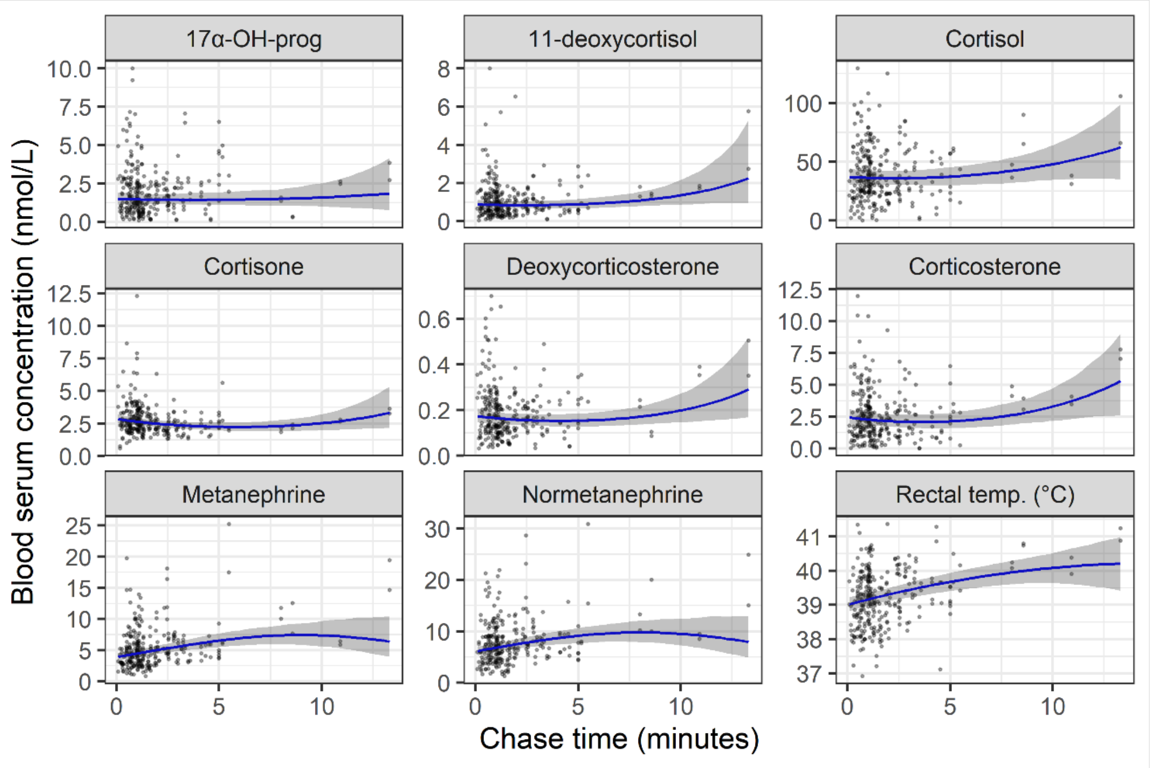

Figure S3. Values of the acute stress response as a function of chase time during captures of female Svalbard reindeer. Blue lines show the predicted response (with 95% CI) for an average individual (6 years old, 4.5 catches in previous years, second catch of the current year) after an average chase time (2 minutes). Points show data, adjusted for individual, chase time, age and year.

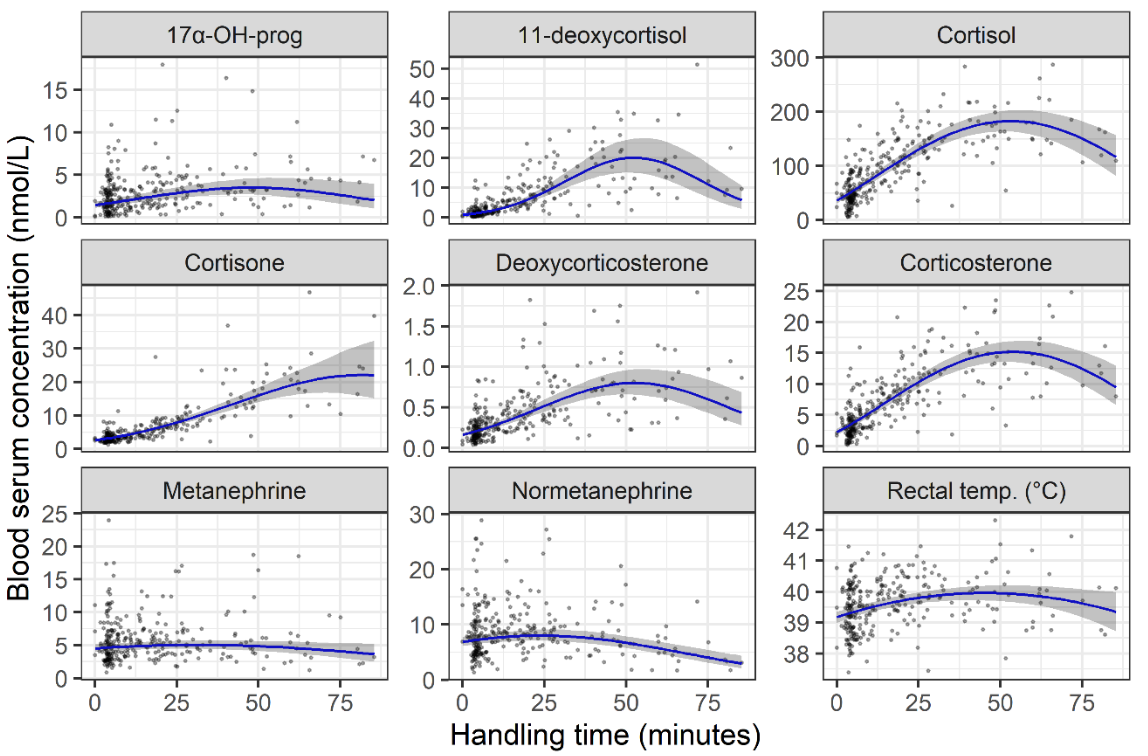

Figure S4. Values of the acute stress response as a function of handling time during captures of female Svalbard reindeer. Blue lines show the predicted response (with 95% CI) for an average individual (6 years old, 4.5 catches in previous years, second catch of the current year) after an average chase time (2 minutes). Points show data, adjusted for individual, chase time, age and year.

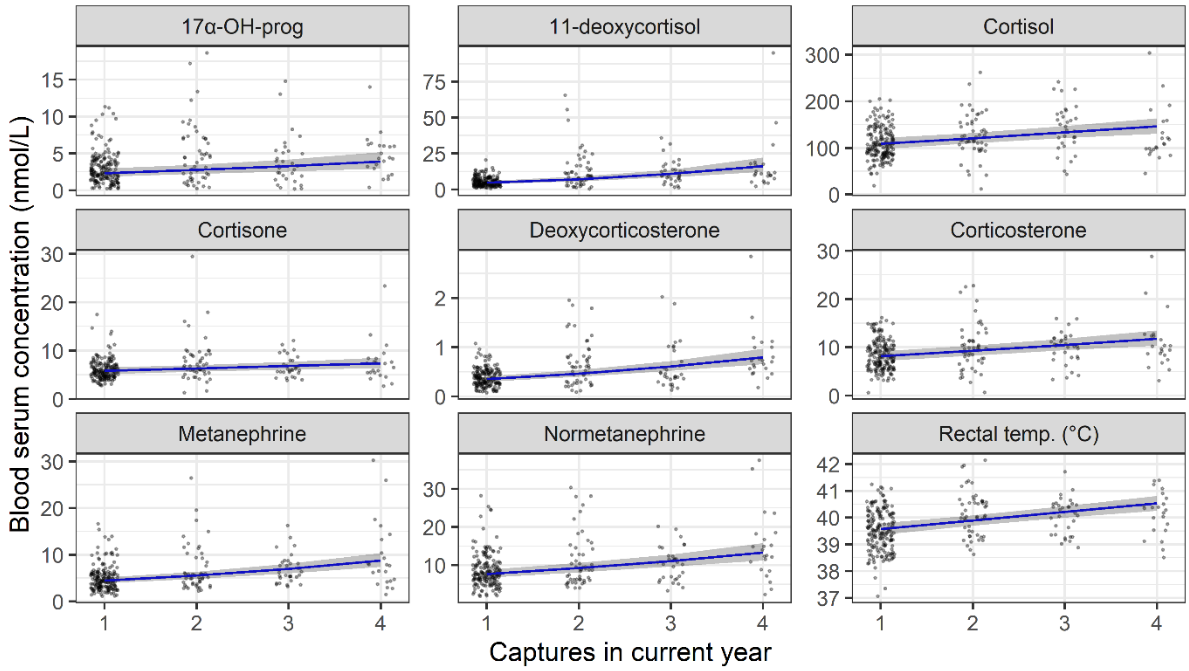

Figure S5. Values of the acute stress response as a function of number of captures the current year for female Svalbard reindeer. Blue lines show the predicted response (with 95% CI) for an average individual (6 years old, 4.5 catch in previous years, second catch of the current years) after an average chase time (2 minutes). Points show data, adjusted for individual, chase time, age, year and catch number.

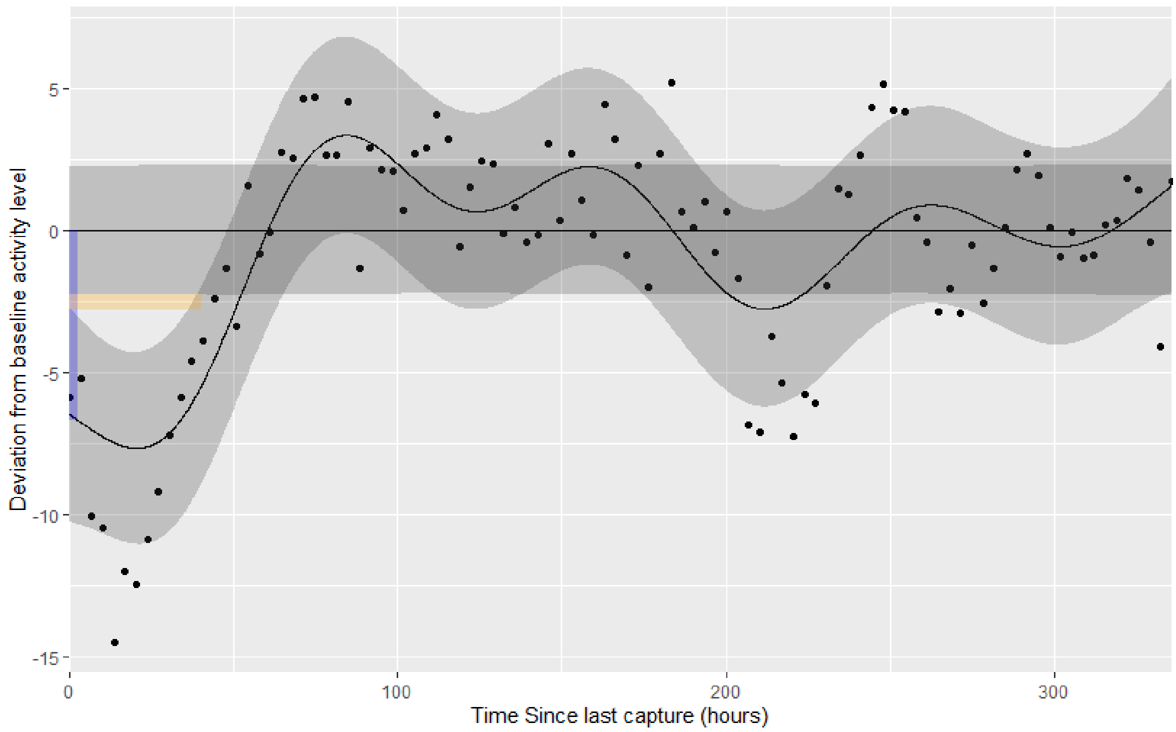

Figure S6. Example of return to baseline (individual G149, second capture) in activity. The horizontal black line shows the baseline activity (after correcting for date and time) and its standard error (grey rectangle). The points show the deviation from baseline (residuals), while the spline show the prediction (and SE) of these deviations as a response of time since last capture. The initial deviation is represented by the blue bar while the recovery time is represented by the yellow bar.

4 Supplementary tables

Table S1**.** Summary of sample sizes used in the different data sets in the study. Shown are each section of analyses and the number of individuals being subjected to 0-4 capture events within a given year.

			**Sample sizes**						
					**Capture number**				
**Analysis**	**Period**	**Year of sampling**	**Total data points (n)**	**Unique individuals (N_{id})**	**0**	**1**	**2**	**3**	**4**
Hormone assays	Winter	2018, 2019	280	77	–	89	25	16	11
Post-capture recovery time	Winter	2018	67	21	–	21	18	15	13
Provocation study	Summer	2018, 2019	134	122	101[a]	16	17[b]		
Offspring survival	Summer	2016-2021	181	119	–	161[c]	20[b, d]		
Reproductive success	Summer	1995–2021	1861	488	1006	855	–		

[a]Unmarked individuals and marked but not captured in the same year grouped due to no difference in response. [b]All captured twice or more the same year grouped to increase sample size. [c]17 of these underwent surgery with sedation. [d]13 of these underwent surgery with sedation.

Table S2. Summary of the physiological stress variables in Svalbard reindeer during capture evens.

Variable	Units	Sample 1		Sample 2	
		Mean ± s.d.	Range (min–max)	Mean ± s.d.	Range (min–max)
17α-OH-progesterone	$nmolL^{-1}$	1.97 ± 1.19	0.08–7.30	2.59 ± 1.44	0.25–9.90
11-deoxycortisol	$nmolL^{-1}$	1.64 ± 2.13	0.18–21.00	6.86 ± 4.34	0.34–22.00
Cortisol	$nmolL^{-1}$	49.21 ± 29.65	8.00–196.00	112.36 ± 39.72	20.00–266.00
Cortisone	$nmolL^{-1}$	3.24 ± 1.50	1.60–14.00	11.41 ± 10.65	2.10–58.00
Deoxycorticosterone	$nmolL^{-1}$	0.21 ± 0.14	0.05–1.30	0.45 ± 0.25	0.10–1.50
Corticosterone	$nmolL^{-1}$	3.40 ± 2.39	0.59–18.00	9.01 ± 4.01	1.10–24.00
Metanephrine	$nmolL^{-1}$	5.16 ± 2.84	1.20–20.00	5.09 ± 2.95	0.75–24.00
Normetanephrine	$nmolL^{-1}$	8.60 ± 4.99	1.50–37.00	8.10 ± 4.08	0.75–23.00
Rectal temperature	°C	39.11 ± 0.76	37.20–41.50	39.61 ± 0.77	37.70–41.40

Table S3. Summary of variance explained by each explanatory variable (columns) for each response variable (rows), extracted from a hierarchical multivariate model (see methods for further details).

Variable	ID	Control	Sedation	Chase	Handling	Capture number	Residual
17α-OH-progesterone	0.21	0.06	0.02	0.00	0.04	0.02	0.65
11-deoxycortisol	0.10	0.02	0.04	0.01	0.39	0.07	0.39
Cortisol	0.09	0.03	0.04	0.00	0.37	0.03	0.43
Cortisone	0.03	0.02	0.04	0.01	0.65	0.03	0.22
Deoxycorticosterone	0.14	0.01	0.05	0.01	0.21	0.07	0.52
Corticosterone	0.10	0.01	0.04	0.01	0.38	0.03	0.43
Metanephrine	0.19	0.02	0.08	0.04	0.01	0.09	0.58
Normetanephrine	0.16	0.06	0.11	0.03	0.04	0.08	0.53
Rectal temperature	0.13	0.02	0.00	0.03	0.04	0.11	0.66

Table S4. Summary of recovery times and Initial deviations for activity (ACT) and heart rate (HR) in female Svalbard reindeer subjected to repeated capture and handling (up to four times) within a six-week period. Deviations are calculated as the Initial deviation divided by the estimated baseline value. Shown are the response types, capture number, mean ± s.d. of the Initial deviation (in % of baseline), mean ± s.d. of the recovery time, the number of individuals investigated for each capture event (n). Not all individuals showed deviation from Initial baseline. In parentheses following the initial deviation and recovery times are the number of individuals whose response was *not* equal to zero.

**Response**	**Capture number**	**Initial deviation (%)**	**Recovery time (h)**	**n**
ACT	1	-10.3 ± 21.7 (5)	10.6 ± 21.9 (6)	23
ACT	2	41.0 ± 32.1 (16)	3.2 ± 3.5 (12)	18
ACT	3	3.8 ± 9.7 (10)	1.9 ± 7.2 (1)	15
ACT	4	7.0 ± 6.7 (9)	0.0 ± 0.0 (0)	13
HR	1	-1.4 ± 11.0 (12)	2.1 ± 5.7 (3)	21
HR	2	23.0 ± 32.3 (16)	11.1 ± 11.6 (13)	18
HR	3	8.4 ± 9.7 (13)	16.1 ± 21.3 (6)	15
HR	4	25.4 ± 42.7 (13)	23.8 ± 22.7 (11)	13

Table S5. Akaike's information criteria for small sample size (AICc) model selection with Initial deviation in activity levels, as percentage of the individuals' baseline level, following captures of female Svalbard reindeer, as response variable. Models were fitted as linear mixed-effects regressions with individual as a random effect using maximum likelihood. The table summarizes the alternative model formulas (explanatory variables), the number of estimated parameters for each model (K), delta (Δ) AICc and the AIC weights (*w*) of each model.

Formula	K	ΔAICc	w
**CapNo + Sedation**	**5**	**0.00**	**1.00**
Sedation	4	23.25	0.00
Intercept	3	49.86	0.00
Captures current year	4	50.80	0.00
Pregnancy status	4	50.95	0.00
Age	4	51.65	0.00
CapLife (incl. this year)	4	51.68	0.00
Pregnancy status + CapNo	5	51.94	0.00
CapOld	4	52.07	0.00
Age + CapNo	5	52.66	0.00

Table S6. Summary of model output of the best model explaining Initial deviation in activity levels, as percentage of the individuals' baseline level, following captures of female Svalbard reindeer. Explanatory variables are whether animals were sedated (Sedation, factor) and capture number (CapNo). The model was fitted using restricted maximum likelihood. Bold text indicates significant effects ($p < 0.05$).

	Initial deviation		
Predictors	Estimates	CI	p
(Intercept)	71.19	51.70 – 90.67	**<0.001**
Sedation [1]	-68.00	-83.67 – -52.33	**<0.001**
CapNo	-17.73	-24.30 – -11.16	**<0.001**
**Random Effects**			
σ^2	399.03		
$\tau_{00\ ID}$	0.00		
ICC	0.00		
N_{ID}	23		
Observations	69		
Marginal R^2 / Conditional R^2	0.54 / 0.54		

Table S7. Akaike's information criteria for small sample size (AICc) model selection with recovery time of activity level, following captures of female Svalbard reindeer, as response variable. Models were fitted as linear mixed-effects regressions with individual as a random effect using maximum likelihood. The table summarizes the alternative model formulas (explanatory variables), the number of estimated parameters for each model (K), delta (Δ) AICc and the AIC weights (w) of each model. Models indicated in bold text are considered most parsimonious. Models indicated in bold text are considered most parsimonious.

Formula	K	Delta_AICc	w
CapNo + Initial deviation	5	0.00	0.19
**Sedation**	**4**	**0.21**	**0.17**
**CapNo**	**4**	**0.68**	**0.13**
CapNo + sedation	5	1.73	0.08
Sedation + Initial deviation	5	2.19	0.06
Pregnancy status + CapNo + Initial deviation	6	2.21	0.06
CapNo + Initial deviation + sedation	6	2.35	0.06
Age + CapNo + Initial deviation	6	2.36	0.06
Pregnancy status + CapNo	5	2.95	0.04
Initial deviation	4	2.96	0.04
Age + CapNo	5	3.00	0.04
Intercept	3	4.60	0.02
CapLife	4	4.77	0.02
CapOld	4	6.63	0.01
Pregnancy status	4	6.81	0.01
Age	4	6.85	0.01

Abbreviations: **CapNo –** captures in current year. **CapLife** – lifetime captures incl. current year. **CapOld** – lifetime captures in past years.

Table S8. Summary of model output of the two equally competing models explaining recovery time activity levels, following captures of female Svalbard reindeer. Explanatory variables are whether animals were sedated (sedation, factor) and capture number (CapNo), contained in two separate models. Final models were fitted with restricted maximum likelihood. Bold text indicates significant effects ($p < 0.05$).

	**Recovery time ~ cap. no**			**Recovery time ~ Sedation**		
Predictors	Estimates	CI	p	Estimates	CI	p
(Intercept)	12.79	5.56 – 20.03	**0.001**	1.78	-2.35 – 5.90	0.391
CapNo	-3.63	-6.46 – -0.79	**0.013**			
Sedation				8.91	2.19 – 15.64	**0.011**
**Random Effects**						
σ^2	165.63			164.63		
$\tau_{00\ ID}$	12.56			12.32		
N_{ID}	23			23		
Observations	69			69		
Marginal R^2 / Conditional R^2	0.091 / NA			0.096 / NA		

Table S9. Akaike's information criteria for small sample size (AICc) model selection with Initial deviation in heart rates, as percentage of the individuals' baseline level, following captures of female Svalbard reindeer, as response variable. Models were fitted as linear mixed-effects regressions with individual as a random effect using maximum likelihood. The table summarizes the alternative model formulas (explanatory variables), the number of estimated parameters for each model (K), delta (Δ) AICc and the AIC weights (w) of each model. Model indicated in bold text is considered most parsimonious.

Formula	K	ΔAICc	w
**Sedation**	**4**	**0.00**	**0.51**
CapNo + sedation	5	2.34	0.16
CapNo	4	3.41	0.09
Pregnancy status + CapNo	5	3.47	0.09
Age + CapNo	5	4.08	0.07
Intercept	3	6.23	0.02
Pregnancy status	4	6.56	0.02
CapLife	4	6.57	0.02
Age	4	7.05	0.01
CapOld	4	8.42	0.01

Abbreviations: **CapNo –** captures in current year. **CapLife** – lifetime captures incl. current year. **CapOld** – lifetime captures in past years.

Table S10. Summary of model output of the best model explaining Initial deviation in activity levels, as percentage of the individuals' baseline level, following captures of female Svalbard reindeer. Explanatory variables are whether animals were sedated (factor) and capture number. The model was fitted using restricted maximum likelihood. Bold text indicates significant effects ($p < 0.05$).

	**Initial deviation**		
Predictors	*Estimates*	*CI*	*p*
(Intercept)	8.20	4.26 – 12.15	**<0.001**
Sedation	-8.73	-15.61 – -1.86	**0.014**
**Random Effects**			
σ^2	171.89		
$\tau_{00\ ID}$	0.30		
ICC	0.00		
N_{ID}	22		
Observations	67		
Marginal R^2 / Conditional R^2	0.090 / 0.092		

Table S11. Akaike's information criteria for small sample size (AICc) model selection with recovery time of heart rates, following captures of female Svalbard reindeer, as response variable. Models were fitted as linear mixed-effects regressions with individual as a random effect using maximum likelihood. The table summarizes the alternative model formulas (explanatory variables), the number of estimated parameters for each model (K), delta (Δ) AICc and the AIC weights (w) of each model. Model indicated in bold text is considered most parsimonious.

Formula	K	ΔAICc	w
**CapNo + Initial deviation**	**5**	**0.00**	**0.44**
Age + CapNo + Initial deviation	6	1.96	0.16
Pregnancy status + CapNo + Initial deviation	6	2.01	0.16
CapNo + Initial deviation + sedation	6	2.42	0.13
Sedation + Initial deviation	5	4.88	0.04
CapNo	4	5.77	0.02
Pregnancy status + CapNo	5	6.93	0.01
Age + CapNo	5	7.05	0.01
CapNo + sedation	5	7.70	0.01
Initial deviation	4	9.06	0.00
Sedation	4	9.56	0.00
CapLife	4	11.85	0.00
Intercept	3	19.00	0.00
Pregnancy status	4	20.41	0.00
Age	4	20.52	0.00
CapOld	4	21.19	0.00

Abbreviations: **CapNo –** captures in current year. **CapLife** – lifetime captures incl. current year. **CapOld** – lifetime captures in past years.

Table S12. Summary of model output of the best model explaining recovery time heart rates, following captures of female Svalbard reindeer. Explanatory variables are Initial deviation (% deviation from individual's baseline) and capture number in current year (CapNo). The model was fitted with restricted maximum likelihood. Bold text indicates significant effects ($p < 0.05$).

	Recovery time		
Predictors	*Estimates*	*CI*	*p*
(Intercept)	-3.65	-11.98 – 4.69	0.383
Initial deviation	0.19	0.06 – 0.33	**0.006**
CapNo	5.69	2.37 – 9.00	**0.001**
**Random Effects**			
σ^2	198.06		
$\tau_{00\ ID}$	26.99		
N_{ID}	22		
Observations	67		
Marginal R^2 / Conditional R^2	0.310 / NA		

Table S13. Summary of model output body mass (in kg) in winter in female Svalbard reindeer. Explanatory variables are capture number (CapRep), grouped into captured once (intercept) or captured twice or more, day of year (numeric), pregnancy status (factor, 1 = pregnant, 0 = not pregnant [intercept]), age (numeric) and an interaction between capture number and day of year (DOY). The model was fitted as a linear mixed effects regression with restricted maximum likelihood, with random slopes fitted for each individual as a function of time (DOY|ID). Bold text indicates significant effects ($p < 0.05$).

		**Body mass**	
Predictors	*Estimates*	*CI*	*p*
(Intercept)	67.30	55.18 – 79.42	**<0.001**
CapRep[2+]	1.87	-9.07 – 12.80	0.733
Day of year	-0.19	-0.29 – -0.08	**0.001**
Pregnancy status [1]	1.45	-0.35 – 3.24	0.112
Age	0.60	-0.17 – 1.36	0.125
CapRep[2+] × DOY	-0.05	-0.16 – 0.06	0.338
**Random Effects**			
σ^2	1.30		
$\tau_{00\ ID}$	21.16		
$\tau_{11\ ID.\ day}$	0.00		
$\rho_{01\ ID}$	-0.77		
ICC	0.87		
N_{ID}	50		
Observations	114		
Marginal R^2 / Conditional R^2	0.510 / 0.936		

Table S14. Akaike's information criteria for small sample size (AICc) model selection with offspring survival in summer female Svalbard reindeer, who were pregnant the previous winter. Each model was fitted with a generalized logistic mixed effects model with individual fitted and year as random intercepts), and an age group predictor variable as a covariate. The table summarizes the alternative model formulas (explanatory variables), the number of estimated parameters for each model (K), delta (Δ) AICc and the AIC weights (w) of each model. Explanations: CapRep – captured once, or more than once (2+) in the same year, fitted as a factor. CapOld – lifetime captures in past years. Sedation – whether the reindeer was sedated and underwent surgery in winter (factor). Body mass (kg) measured in previous winter. Model indicated in bold text is considered most parsimonious.

Formula	K	ΔAICc	w
**Body mass × CapRep**	**9**	**0.00**	**0.42**
Body mass × CapRep + Sedation	10	0.69	0.30
Body mass × (CapRep + Sedation)	11	2.93	0.10
Body mass	7	4.62	0.04
Body mass × Sedation × CapRep	13	5.11	0.03
Body mass Sedation	9	5.85	0.02
Body mass + Sedation	8	5.86	0.02
Body mass + CapOld	8	5.90	0.02
Body mass + CapRep	8	6.78	0.01
Body mass + Sedation + CapRep	9	7.73	0.01
Body mass × Sedation + CapRep	10	7.74	0.01
Body mass × CapOld	9	8.06	0.01
Intercept	6	12.03	0.00
CapRep	7	14.11	0.00

Table S15. Summary of model output of the most parsimonious model explaining offspring survival in summer of female Svalbard reindeer, who were pregnant the previous winter. Explanatory variables are body mass measured in winter, whether the reindeer were captured once (intercept) or more than once (2+) in winter (CapRep), age class in winter (factor; intercept is class 1-3). Body mass was scaled to a mean of 0 and standard deviation equal to 1 (mean of 52.7±4.97 kg). The model was fitted as a generalized linear mixed regression with a binomial logit function. Bold text indicates significant effects ($p < 0.05$).

	**Calf at heel**		
Predictors	*Odds Ratios*	*CI*	*p*
(Intercept)	2.70	0.96 – 7.65	0.061
Body mass	2.40	1.43 – 4.02	**0.001**
CapRep [2+]	2.16	0.40 – 11.52	0.368
Age class [4-8]	1.10	0.39 – 3.14	0.852
Age class [9-11]	1.08	0.30 – 3.85	0.902
Age class [12-15]	0.83	0.18 – 3.92	0.819
Body mass × CapRep [2+]	0.12	0.02 – 0.60	**0.010**
**Random Effects**			
σ^2	3.29		
$\tau_{00\ ID}$	0.10		
$\tau_{00\ year}$	0.28		
N_{ID}	119		
N_{year}	6		
Observations	181		
Marginal R^2 / Conditional R^2	0.18 / 0.27		

Table S16. Akaike's information criteria for small sample size (AICc) model selection with reproductive success in summer in female Svalbard reindeer. Each model was fitted with a generalized additive mixed effects model with individual fitted and year as random intercepts (intercept model). The table summarizes the alternative model formulas (explanatory variables), the degrees of freedom (df) for each model, delta (Δ) AICc and the AIC weights (w) of each model. Explanations: Captured – captured zero times or once in the same year, fitted as a factor. S() – smooth term. Mass - Average body mass of adult females in April. Model indicated in bold text is considered most parsimonious.

Formula	df	ΔAICc	W
**s(Mass) × Captured**	**3.9**	**0.00**	**0.77**
s(Mass)	3.9	3.60	0.13
intercept	1.0	5.53	0.05
s(Mass) + Captured	4.9	5.73	0.04
Captured	2.0	7.67	0.02

Table S17. Summary of model output of reproductive success of female Svalbard reindeer between 1995 and 2021. Explanatory variables are the annual average body mass in winter (mass), whether the reindeer were not captured (0) or captured once (1) (Captured), and an interaction between capture number and April body mass. The model was fitted as a generalized additive regression with a binomial logit function. For the smooth terms, estimated degrees of freedom (edf) and significance of each term are provided. S() – smooth term. April body mass was scaled to a mean of 0 and standard deviation equal 1 (mean of 52.1±3.24 kg). Year and ID are fitted as random intercepts. Bold text indicates significant effects ($p < 0.05$).

	Calf at heel		
Predictors	*Odds ratios*	*CI*	*p*
(Intercept)	1.45	1.04 – 2.02	**0.029**
		Edf	*p*
S(mass) × Captured [0]		2.92	**0.002**
S(mass) × Captured [1]		2.16	**0.007**
S(year)		21.14	**<0.001**
S(ID)		47.88	**0.015**
Observations	1861		
Adj. R^2	0.16		

Table S18. Akaike's information criteria for small sample size (AICc) model selection with alert distances (in meters) in female Svalbard reindeer being approached by humans on foot as the response variable. Each model contained the log-transformed encounter (start) distance as a control. The table summarizes the alternative model formulas (explanatory variables), the number of estimated parameters for each model (K), delta (Δ) AICc and the AIC weights (w) of each model. Explanations: CapRep – grouping variable for individuals having been caught zero (0), once (1) or more than once (2+) in March/April the current year, f() – factor variable (otherwise numeric), year – year of study (2018/2019), calf – calf at heel, CapLife – total lifetime captures, sedation – animal was sedated and underwent surgery during capture. All models are fitted as linear regressions. Model indicated in bold text is considered most parsimonious.

Formula	K	ΔAICc	w
**ln(Start)**	**3**	**0.00**	**0.29**
ln(Start) + f(Year)	4	0.97	0.18
ln(Start) + CapLife	4	1.60	0.13
ln(Start) + f(Sedation)	4	1.97	0.11
ln(Start) + f(Calf)	4	2.08	0.10
ln(Start) + f(Year) + f(CapRep)	6	3.64	0.05
ln(Start) + CapLife + f(Calf)	5	3.71	0.04
ln(Start) + f(CapRep)	5	3.81	0.04
ln(Start) + f(Sedation) + f(Calf)	5	4.06	0.04
ln(Start) + f(CapRep) + f(Calf)	6	5.94	0.01
ln(Start) + f(Sedation) + f(CapRep)	6	5.98	0.01
ln(Start) + f(CapRep) × f(Calf)	8	8.90	0.00
Intercept	2	73.26	0.00

Table S19. Akaike's information criteria for small sample size (AICc) model selection with flight initiation distances (in meters) in female Svalbard reindeer being approached by humans on foot as the response variable. Each model contained the log-transformed encounter (start) distance as a control. The table summarizes the alternative model formulas (explanatory variables), the number of estimated parameters for each model (K), delta (Δ) AICc and the AIC weights (w) of each model. Explanations: CapRep – grouping variable for individuals having been caught zero (0), once (1) or more than once (2 +) in March/April the current year, f() – factor variable (otherwise numeric), year – year of study (2018/2019), calf – calf at heel, CapLife – total lifetime captures, sedation – animal was sedated and underwent surgery during capture. All models are fitted as linear regressions. Model indicated in bold text is considered most parsimonious.

Formula	K	ΔAICc	w
ln(Start) + CapLife	4	0.00	0.20
**ln(Start)**	**3**	**0.43**	**0.16**
ln(Start) + CapLife + f(Calf)	5	0.70	0.14
ln(Start) + f(Sedation)	4	1.16	0.11
ln(Start) + f(Calf)	4	1.16	0.11
ln(Start) + f(Sedation) + f(Calf)	5	1.57	0.09
ln(Start) + f(Year)	4	2.42	0.06
ln(Start) + f(CapRep)	5	3.08	0.04
ln(Start) + f(CapRep) + f(Calf)	6	3.53	0.03
ln(Start) + f(Sedation) + f(CapRep)	6	5.23	0.01
ln(Start) + f(Year) + f(CapRep)	6	5.27	0.01
ln(Start) + f(CapRep) × f(Calf)	8	5.30	0.01
Intercept	2	29.58	0.00

Table S20. Akaike's information criteria for small sample size (AICc) model selection with comfort distances (in meters) in female Svalbard reindeer being approached by humans on foot as the response variable. Each model contained the log-transformed encounter (start) distance as a control. The table summarizes the alternative model formulas (explanatory variables), the number of estimated parameters for each model (K), delta (Δ) AICc and the AIC weights (w) of each model. Explanations: CapRep – grouping variable for individuals having been caught zero (0), once (1) or more than once (2 +) in March/April the current year, f() – factor variable (otherwise numeric), year – year of study (2018/2019), calf – calf at heel, CapLife – total lifetime captures, sedation – animal was sedated and underwent surgery during capture.

Formula	*K*	ΔAICc	*w*
**ln(Start) + f(CapRep) + f(Calf)**	**6**	**0.00**	**0.48**
ln(Start) + f(Sedation) + f(Calf)	5	2.36	0.15
ln(Start) + CapLife + f(Calf)	5	2.47	0.14
ln(Start) + f(CapRep) × f(Calf)	8	3.20	0.10
ln(Start) + f(CapRep)	5	5.28	0.03
ln(Start) + CapLife	4	5.97	0.02
ln(Start) + f(Calf)	4	6.12	0.02
ln(Start) + f(Year) + f(CapRep)	6	6.42	0.02
ln(Start) + f(Sedation)	4	6.96	0.01
ln(Start) + f(Sedation) + f(CapRep)	6	7.35	0.01
ln(Start) + f(Year)	4	8.45	0.01
ln(Start)	3	9.33	0.00
Intercept	2	16.01	0.00

Table S21. Summary of model output of comfort distances of female Svalbard reindeer being approached by humans on foot as the response variable. Explanatory variables are capture number, grouped into captured once (intercept) or captured twice or more, day of year (numeric), calf (factor, 1 = with a calf at heel, 0 = no calf at heel [intercept]. The model was fitted with restricted maximum likelihood. Bold text indicates significant effects ($p < 0.05$).

	**Comfort distance**		
Predictors	*Estimates*	*CI*	*p*
(Intercept)	-112.35	-250.36 – 25.66	0.110
ln(start)	42.42	15.71 – 69.13	**0.002**
CapRep [1]	25.61	-2.99 – 54.20	0.079
CapRep [2+]	42.49	13.93 – 71.05	**0.004**
Calf [1]	26.20	7.14 – 45.25	**0.007**
Observations	129		
R^2 / R^2 adjusted	0.17 / 0.15		
